# “Omics” data integration and functional analyses link Enoyl-CoA hydratase, short chain 1 to drug refractory dilated cardiomyopathy

**DOI:** 10.1186/s12920-018-0439-6

**Published:** 2018-12-12

**Authors:** Nzali V. Campbell, David A. Weitzenkamp, Ian L. Campbell, Ronald F. Schmidt, Chindo Hicks, Michael J. Morgan, David C. Irwin, John J. Tentler

**Affiliations:** 1 Colorado Clinical and Translational Sciences Institute, Mail Stop B141, 12401 E. 17th Ave, Aurora, CO 80045 USA; 2 Anschutz Medical Campus, University of Colorado, 13001 E 17th Pl, Aurora, CO 80045 USA; 3 Stanford University, 450 Serra Mall, Stanford, 94305 CA USA; 4 Schmidt Motors, 501 W 1st St, Ogallala, NE 69153 USA; 5 Louisiana State University Health Sciences Center, New Orleans, LA 70112 USA; 6 28998 Road N, Dolores, 81323 CO United States

**Keywords:** drDCM, Omics, RNA-Seq, IPA, ECHS1, DBT, MIR3944, rs10466126, BCAA, Mitochondria

## Abstract

**Background:**

Large-scale “omics” datasets have not been leveraged and integrated with functional analyses to discover potential drivers of cardiomyopathy. This study addresses the knowledge gap.

**Methods:**

We coupled RNA sequence (RNA-Seq) variant detection and transcriptome profiling with pathway analysis to model drug refractory dilated cardiomyopathy (drDCM) using the BaseSpace sequencing hub and Ingenuity Pathway Analysis. We used RNA-Seq case-control datasets (*n* = 6 cases, *n* = 4 controls), exome sequence familial DCM datasets (*n* = 3 Italians, *n* = 5 Italians, n = 5 Chinese), and controls from the HapMap project (n = 5 Caucasians, and n = 5 Asians) for disease modeling and putative mutation discovery. Variant replication datasets: *n* = 128 cases and *n* = 15 controls. Source of datasets: NCBI Sequence Read Archive. Statistics: Pairwise differential expression analyses to determine differentially expressed genes and t-tests to calculate *p*-values. We adjusted for false discovery rates and reported q-values. We used chi-square tests to assess independence among variables, the Fisher’s Exact Tests and overlap p-values for the pathways and p-scores to rank network.

**Results:**

Data revealed that ECHS1(enoyl-CoA hydratase, short chain 1(log_2_(foldchange) = 1.63329) hosts a mirtron, MIR3944 expressed in drDCM (FPKM = 5.2857) and not in controls (FPKM = 0). Has-miR3944-3p is a putative target of BAG1 (BCL2 associated athanogene 1(log_2_(foldchange) = 1.31978) and has-miR3944-5p of ITGAV (integrin subunit alpha V(log_2_(foldchange) = 1.46107) and RHOD (ras homolog family member D(log_2_(foldchange) = 1.28851). There is an association between *ECHS1*:11 V/A(rs10466126) and drDCM (*p* = 0.02496). The interaction (*p* = 2.82E-07) between *ECHS1*:75 T/I(rs1049951) and *ECHS1*:rs10466126 is associated with drDCM (*p* < 2.2e-16). *ECHS1*:rs10466126 and *ECHS1*:rs1049951 are in linkage disequilibrium (D’ = 1). The interaction (*p* = 7.84E-08) between *ECHS1*:rs1049951 and the novel *ECHS1*:c.41insT variant is associated with drDCM (p < 2.2e-16). The interaction (*p* = 0.001096) between *DBT* (Dihydrolipoamide branched chain transacylase E2):384G/S(rs12021720) and *ECHS1*:rs10466126 is associated with drDCM (p < 2.2e-16). At the mRNA level, there is an association between ECHS1 (log_2_(foldchange) = 1.63329; q = 0.013927) and DBT (log_2_(foldchange) = 0.955072; q = 0.0368792) with drDCM. ECHS1 is involved in valine (−log (*p* = 3.39E00)), isoleucine degradation (*p* = 0.00457), fatty acid β-oxidation (−log(p) = 2.83E00), and drug metabolism:cytochrome P450 (z-score = 2.07985196) pathways. The mitochondria (−log(p) = 8.73E00), oxidative phosphorylation (−log(p) = 5.35E00) and TCA-cycle II (−log(p) = 2.70E00) are dysfunctional.

**Conclusions:**

We introduce an integrative data strategy that considers the interplay between the DNA, mRNA, and associated pathways, which represents a possible diagnostic, prognostic, biomarker, and personalized treatment discovery approach in genomically heterogeneous diseases.

**Electronic supplementary material:**

The online version of this article (10.1186/s12920-018-0439-6) contains supplementary material, which is available to authorized users.

## Background

At present, biventricular or left ventricular dilation and systolic dysfunction with no abnormal hypertension and valve disease or coronary artery disease enough to produce global systolic malfunction characterize dilated cardiomyopathy (DCM: OMIM 115200). Causative factors can be genetic or non-genetic, and, sometimes, genetic predispositions can interact with environmental factors to trigger DCM. On the other hand, non-genetic causes include drugs and toxins, myocarditis, and peripartum cardiomyopathy. However, a combination of the factors mentioned above can also cause DCM [1–4]. Dissecting the molecular mechanisms underlying the disease remains a challenge and regrettably, the end-point is usually end-stage drug-refractory heart failure and heart transplantation [5]. Advances in technology have expanded the availability of omics data. The goal of these data is to determine models that predict outcomes and the phenotype, which expose biomarkers and give insight into the genomic foundation of how complex traits are inherited. However, robust strategies to tie together and use these data to discover valid connections are lacking [6]. Approaches that integrate “omics” data link the gap between assessing variation at only one stage of regulation of the central dogma and understanding the underlying complexity within biological systems. Understanding such complexity requires intricate models, which consider variation across various stages of biological regulation. Data coupling identifies important genomic factors and connections that elucidate or predict risk factors for disease and other biological outcomes. It provides an avenue to making sense of and appreciating the greater complexity that underlies human disease [6].

Accomplishments in revealing the genomic and genetic pathogenic architecture of complex traits have been meek, in part because of limited investigations that consider variation interplay across each stage of the central dogma [6]. In their 2017 review, Hang et al. presented three methods used to integrate multiple omics data. They outlined how comprehensive tools and multi-omic data integration have advanced. They discuss three main algorithms used, unsupervised, supervised and semi-supervised data integration methods [7].

Briefly, unsupervised data integration draws inferences from unlabeled response variable input datasets. It uses several methods to investigate their biological profiles to allocate objects into various clusters or subgroups. Conversely, the supervised integrative approach considers the subject phenotype, diseased or healthy and uses machine training omics datasets in their analyses. This approach uses the biological information of the labeled objects to get patterns for diverse phenotypes and allocate labels to unlabeled data by comparing the models. Semi-supervised integrative methods, interface unsupervised and supervised approaches. They incorporate labeled and unlabeled samples to create learning algorithms. They mostly build object-wise similarity networks by gathering omics data and assigning labels to unknown objects through their associations to the labeled objects [7].

In our integrative approach, one does not use unlabeled response variables, training datasets, or create learning algorithms to draw inferences. Here, we present our conceptual and analytical models used in our integrative method, based on both the dominant and alternative paradigms, [6] and approaches coined by Vanderweele and Robins [8].

In this report, we aimed to use our methods to provide strategies and insight that can be exploited to reveal mutations, modifier genes, pathogenic pathways, and to model complex traits. We posited that our omics integrative method would be able to model molecular processes that lead to drug refractory DCM (drDCM) and discriminate mutations from modifiers, pathogenic pathways from those that alter the outcome. We report a novel approach for omics data integration that considers the interplay between DNA, mRNA and pathway analyses. The methods can be used to model complex traits while illuminating genomic biomarkers and targets for therapeutic intervention. Our technique revealed that the *ECHS1* gene harbors a V11A (rs10466126) putative mutation in the first exon, and two presumed modifiers, T75I (rs1049951) and a novel variant *ECHS1*:c.41insT in the second exon. Additionally, the *ECHS1* gene hosts a mirtron, MIR3944 in the first intron that is only expressed in drDCM cases and not controls. This microRNA is a putative target of BAG1, ITGAV, and RHOD. Patients with drDCM have genomic insults in the mitochondria that are intertwined with environmental factors, branched-chain amino acids (BCAAs), and long and very long chained fatty acids (LVLCFAs) in the pathogenesis of drDCM. Interactions between errors in the patients’ genomic makeup and breakdown of BCAAs and LVLCFAs lead to cardiotoxicity, dysfunction of the mitochondria, oxidative phosphorylation and the TCA (tricarboxylic acid) cycle.

## Methods

### Conceptual framework

**Fig. 1:**
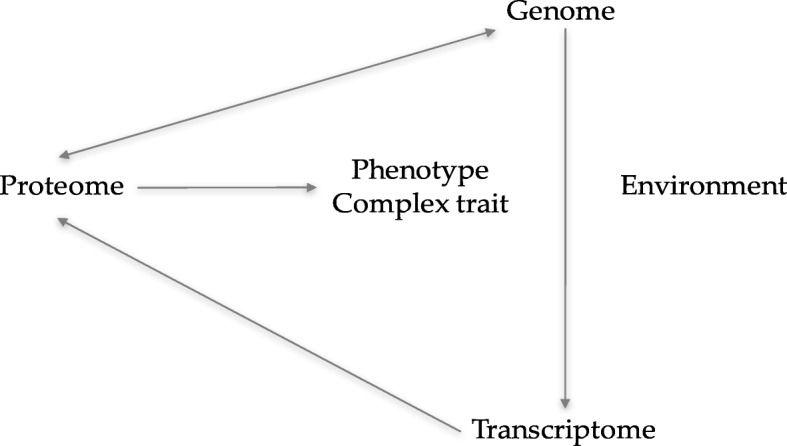
Conceptual Model

Our conceptual model (Fig. 1) utilizes the dominant paradigm that dissimilarity at the genomic step will lead to changes in gene expression. Changed gene expression would, in turn, lead to protein alterations, followed by disease. However, the model also considers the alternative probability that many levels of molecular and environmental dissimilarities add to disease risk in a convoluted nonlinear, and interactive fashion [6]

.

We defined data integration as merging genomics and transcriptome data, pathway, functional, and association analyses. The data are the predictor variables that make it possible for one to have a more systematic and complete modeling of a complex trait. The complex phenotype, here drDCM, might arise because of interactions among biological variations at many stages of genomic control. However, the disease might also occur because of one’s environment.

### Analytical model

#### Statistical measures

The term effect modification is used by epidemiologists to infer that the effect of one variable on another change across the strata of a third. There are numerous ways to measure the effect and several processes by which variables can be effect modifiers for the association between cause and an effect. Assessment of effect alteration is determined by the odds ratio, which measures relationships between exposure and an outcome in a case-control study. The risk difference estimates departures from the additivity of effects. The risk ratio also called a relative risk evaluates the quotient of the chance of an event happening in a group that is exposed to the probability of the incident arising in an entity that is not exposed [8–11].

We have constructed our analytical model, in part on the causal risk difference or causal odds ratio, as a measure of choice measures introduced by Vanderweele and Robins in 2007 [8] although, in our model, we have used the chi-square statistic to assess independence or associations. By doing so, the relationships an effect modifier may have on the variable creating the cause and the variable generating the effect are explicit. The model also operates under the knowledge that effect modification evaluated on the causal risk difference scale falls under the bigger picture of interaction [8]. In considering variables that may be confounders (i.e., conditioning on variables that are in between the exposure (mutation) and the outcome variable (DCM)), we consider effect modifiers that are not a result of the exposure variable under study. The effect modifier is a differentially expressed gene carrying a variant that its interaction with other variations is statistically associated with the outcome.

#### Statistical associations

One can demonstrate mathematical relations on causal directed acyclic graphs in numerous ways. Two variables can be statistically connected if one (e.g., mutation) is a direct or indirect cause of the other (e.g., DCM) or vice versa. However, even though neither is the cause of the other, there can be a statistically significant connection between the mutation and DCM if they have some common trigger. There can also be an association between a variation and DCM if they have a common outcome (for example heart failure) and if the relationship is assessed within the strata of heart failure. In other words, a mutation and DCM will, in general, be statistically associated given heart failure, if heart failure is a shared result of the variation and DCM [12].

Thus, statistical associations between variables can be determined by blocked and unblocked nodes in a series that are linked by edges regardless of arrowhead direction (a path). A direct route follows the edges in the path denoted by the graph’s arrows. A collider is a type of a node found on a path that both the edge from and to that node have arrowheads into the node. A path between a mutation and DCM, for example, is said to be blocked given some set of variables, say ***C***, if either there is a variable in ***C*** on the path that is not a collider. One can also say that the route is impassable if there is a collider on the way such that neither the collider itself nor any of its progenies are in ***C***. If all paths between a mutation and DCM are blocked given ***C***, then the variation and DCM are conditionally independent given ***C*** [13].

#### Effect modification, a structural classification

**Fig. 2:**
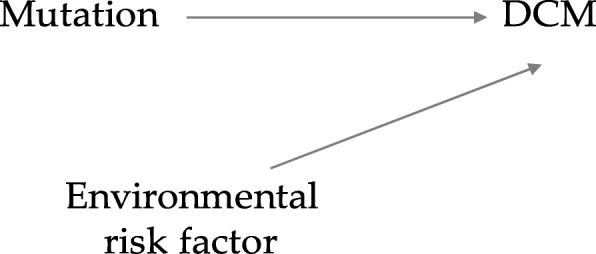
Direct Effect Modification

To consider what type of associations effect modifiers may show concerning the variable creating the cause and the variable generating the effect, we use causal directed acyclic graphs. Using this approach produces a classification of the diverse kinds of effect modification [8]. For example, Fig. 2 shows no shared causes of the mutation (exposure) and the environmental risk factor (effect modifier). Since both the variation and the environmental risk factor influence DCM, the causal relationship between these variables can be illustrated in the context of a causal directed acyclic graph (Fig. 2). Under this model, the environmental risk factor is a direct modifier of the causal effect of the mutation on DCM because the environmental risk factor is an immediate cause of DCM [8]

.

**Fig. 3:**
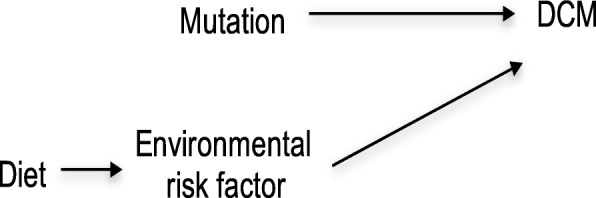
Indirect Effect Modification

**Fig. 4:**
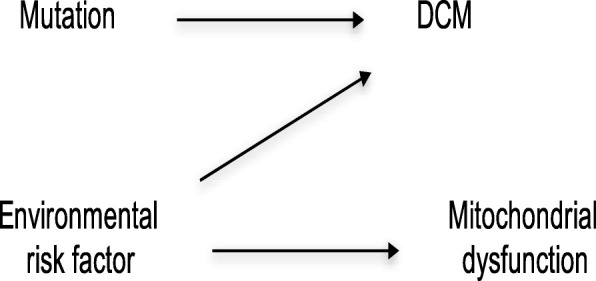
Effect Modification by Proxy

In the next model, we assume information is available for the subject’s diet. We also find that the diet affects the environmental risk factor but does not affect whether the subject gets DCM directly (Fig. 3) [8]. In this case, the patient’s diet will serve as a modifier that alters the effect on the causal risk difference scale for the consequence of the mutation on DCM. This is because conditioning on a diet affects the environmental risk factor, which serves as an effect modifier for the causal effect of the variation on DCM. Diet is then an indirect effect modifier for the causal effect of the mutation on DCM since the diet affects DCM directly through the environmental risk factor [8]. Now suppose the environmental risk factor also determines mitochondrial dysfunction in the patient (Fig. 4). Under this model, mitochondrial dysfunction may serve as an effect modifier on the causal risk difference scale for the effect of the mutation on DCM because conditioning on mitochondrial dysfunction gives information on the environmental risk factor, which acts as an effect modifier for the causal effect of the mutation on DCM. However, since mitochondrial dysfunction is not a direct cause of DCM one would say that mitochondrial dysfunction is an effect modifier of the causal effect of the mutation on DCM by proxy [8]

.

**Fig. 5:**
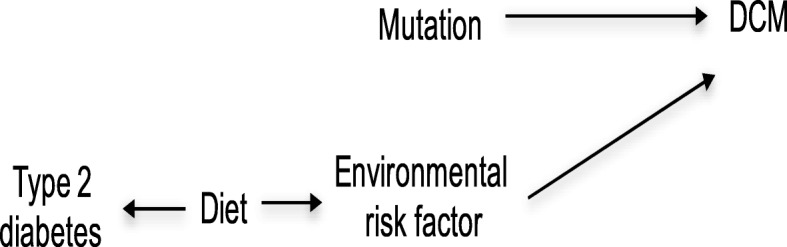
Effect Modification by Common Cause

Finally, suppose additional information on diabetes another disease the subject has existed. The causal connections among the variables could be denoted by the causal directed acyclic graph given in Fig. 5 [8]. Diabetes might act as an effect modifier of the causal risk difference of the mutation on DCM because conditioning on diabetes provides information on the subject’s diet, which affects the environmental risk factor, which acts as an effect modifier for the causal effect of the mutation on DCM. Because the diet is a common cause of the environmental risk factor (that is a direct cause of DCM) and diabetes, which we are conditioning on, one might refer to diabetes as an effect modifier by a common cause of the consequence of the variation on DCM [8]

.

Vanderweele and Robins, 2007 showed that many if not all cases of effect modification can fit into one of the four models presented above. One can distinguish these four models in many ways. The effect modifiers introduced in Figs. 4 and 5, for the effect of a mutation on DCM might not themselves have a causal impact on DCM. However, in the direct and indirect effect modification, the effect modifier does have a causal effect on DCM. Conversely, in the case of effect modification by proxy and by a common cause, the effect modifier does not. In this case, the unblocked path from a set of non-descendants (say ***N***) to the environmental risk factor that gives rise to the required relationship between ***N*** and the ecological risk factor will be a backdoor path from ***N*** to the environmental risk factor [8].

Additionally, one might also differentiate direct effect modification from the other three kinds. If one is conditioning on many variables, which include all the direct effect modifiers (say ***M***), then no other variable on the network will continue to act as an effect modifier for the causal effect of the mutation on DCM. The reason is that ***M*** blocks all paths from any other potential effect modifier ***N*** to DCM. Direct effect modifiers take precedence over different kinds [8].

In their second Theorem, Vanderweele, and Robins, state that if the exposure “is the only variable on the directed acyclic graph which is a direct cause of the outcome, then there can be no variable on the directed acyclic chart which acts as an effect modifier for the relationship between exposure and outcome” [8]. This is because another variable, which could influence the outcome must do so through the exposure. Intervening in the exposure will displace any effect this other variable might otherwise have had [8].

**Fig. 6:**
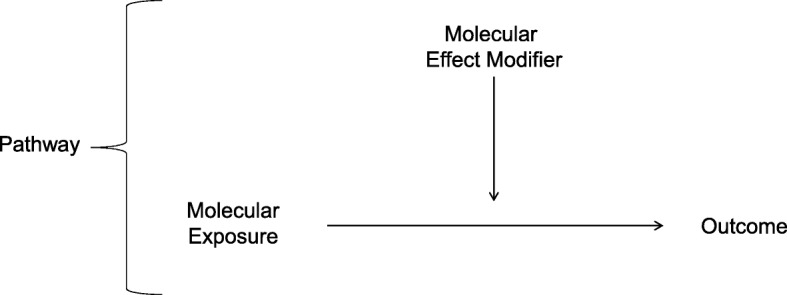
Analytical Model

Our analytical model introduces the novel idea that there are variables on the directed acyclic chart, which act as molecular effect modifiers for the causal association between the exposure and the outcome. In our model (Fig. 6), we hypothesized that within a pathogenic pathway there is at least one mutation within a differentially expressed gene whose causal effect on the outcome is altered by an effect modifier. In this model, there is no direct statistical association between the effect modifiers (i.e., a variant) with the outcome. However, there is a statistical association between interactions among the exposure variables and the effect modifiers, with the outcome.

### Study design

**Fig. 7:**
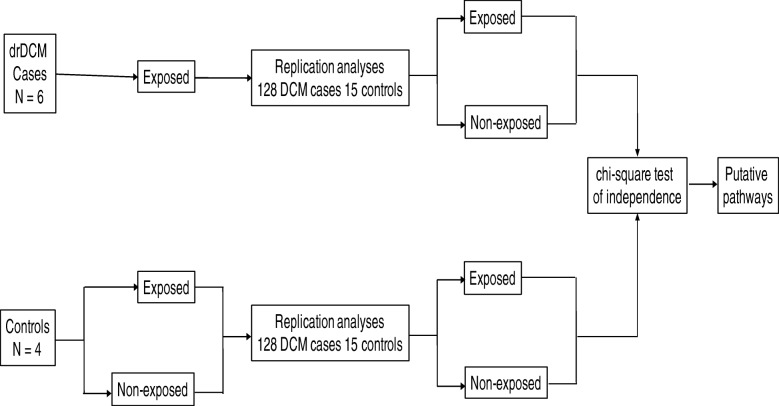
Study design

This study is a quantitative case-control design. We calculated associations between genomic variations and genes over-expressed in pathways to DCM. We used data sets from the left ventricles of hearts from six drDCM cases and four controls without heart disease. We replicated variants shared by all patients, confirmed in exome sequence data sets and discovered in a gene that was differentially expressed and found in an over-represented pathway in 15 controls and 128 DCM cases. We employed the chi-square test of independence to discover interactions and relationships between drDCM and variations or whether there were interactions among the variants (Fig. 7).

To discover putative pathogenic pathways, mutations and effect modifiers, we examined pathways with variants directly associated with drDCM. We defined such pathways as putative pathogenic pathways and the variants as suspected mutations. We described variants that interacted with other variants and whose interactions were linked to drDCM as suspected effect modifiers (Fig. 7).

### Data sets and source

#### Data source

We got all data sets from the sequence read archive (SRA) database [14].

#### Case-control RNA-Seq data sets

The ribonucleic acid sequence (RNA-Seq) data sets for the main study consisted of data from the left ventricles of four heart organs donated for heart transplantation (SRA study accession # SRP052978; at https://www.ncbi.nlm.nih.gov/Traces/study/?acc=SRP052978). The hearts were not suitable for implantation but appropriate for controls. We got six data sets from the left ventricles of patients with drDCM who had a heart transplant [14].

#### Exome sequence data sets from Italian pedigrees

We got three data sets from members of a family from Italy with familial DCM. [15] We collected data from five other individuals of another family with familial DCM from Italy. [15] One subject, in this family was included in our analyses as a control since she did not have DCM. Family members from both datasets were from the Familial Cardiomyopathy Registry (SRA study number: SRP022855; at https://www.ncbi.nlm.nih.gov/Traces/study/?acc=SRP022855) [16]. These two families carried a c.517 T C > T, Arg173Trp TNNT2 mutation [15].

#### Exome sequence data sets from a Chinese pedigree

We obtained data sets from five patients with familial DCM from China (SRA study #SRP066837; at <https://www.ncbi.nlm.nih.gov/Traces/study/?acc=SRP066837&go=go). The samples used were from the Heart Failure Center, Department of Cardiology, Beijing Chao-Yang Hospital, Capital Medical University, 8# Gong-Ti South Road, Beijing, 100,023, in China. The DNA was from blood tissue [14].

#### Control data sets from the HapMap project

We got nine data sets from the HapMap project (Additional file 1: Table S1). Four datasets were from unrelated Caucasians from Utah, and five Asians from China. The Han Chinese were used to match the ethnicity of the Chinese pedigree with drDCM. We used the UTAH/MORMON population because evidence showed inbreeding within families [17] like the breeding patterns seen in one of the Italian pedigree [15].

#### Variant replication RNA-Seq data sets

We utilized a data set generated from 15 controls (SRA Study: SRP041706, SRA Study: SRP093240, SRA Study: SRP021193, and SRA Study: ERP003613) and 128 DCM patients (*SRA Study:* ERP009437) for the variant detection investigates. The datasets included 798 differentially expressed genes, and the variant list had 408 variants at the intersection of all 128 DCM cases.

### Measures

#### Outcome and exposure variables

The primary outcome variable was drDCM, affected or normal. We described variants in pathways associated with drDCM as exposure variables. These included variations directly related to the outcome. DNA changes that were indirectly related to interactions with other variants were defined as putative effect modifiers. Collectively, these variations were classified as the genetic risk factors of drDCM.

#### Inclusion standards for pedigree data sets

We included data from subjects with familial DCM that were sequenced with an Illumina high throughput-sequencing instrument. We included data sets from the Hap Map project to match race (Italy: Caucasian (Utah) and China: Asian)), breeding styles (Italy: inbreeding), and sample sizes (FAM001: 3; FAM027: 5; Chinese: 5) of the pedigree data sets.

#### Inclusion standards for case-control study design data sets

We included data sets sequenced with an Illumina sequencing instrument from unrelated subjects with DCM or drDCM. Control data sets used in the transcriptome analyses originated from the left ventricle of the heart muscle, assessed as healthy and not having heart disease. Data sets from patients were from the left ventricles of the heart. We included data sets from other regions other than the left ventricles of the heart in the variant detection investigations as additional controls.

#### Diagnostic standards for exome sequence pedigree data sets

We utilized the diagnostic criterion for the Italian families set by the original authors [18]. We established the diagnostic measures for the patients from Chinese on information given in the SRA database (https://www.ncbi.nlm.nih.gov/Traces/study/?acc=SRP066837&go=go), saying that the patients had DCM.

#### Diagnostic criteria for subjects in RNA-Seq case-control data sets

We used the diagnostic measures set by the primary investigators to classify the controls. They defined subjects as having healthy hearts (https://www.ncbi.nlm.nih.gov/Traces/study/?acc=SRP052978). The diagnostic or medical criteria or phenotype information for data sets from the Hap Map project was not revealed [19]. We set the diagnostic standards as unknown.

#### Clinical status evaluation

We cataloged data sets from subjects as unknown if we did not have medical or phenotype data and affected when individuals had drDCM or DCM. We categorized data sets from individuals as controls when they did not have heart disease or were described as having healthy hearts by the original researchers.

### Bioinformatics

#### RNA-Seq data analysis

We used apps in the BaseSpace sequencing hub for all bioinformatics analyses (basespace.illumina.com). We imported FASTQ files from the SRA to BaseSpace using the SRA import App. We used the RNA-Seq alignment App for quality control using built-in Apps that include, TopHat version 2.1.0 aligned reads to the reference genome (hg19) and Bowtie aligner version 0.12.9 producing BAM output files. We used the BAM files as input in the Isaac Variant Caller App. The Isaac Variant Caller created VCF files. The RNA-Seq alignment App filters reads that do not pass all the quality checks. It identifies indels and SNPs and computes probabilities of each probable genotype [20]. We used cufflinks to generate the Fragments Per Kilobase of sequence per Million mapped reads (FPKM) output files. (basespace.illumina.com).

We also utilized the Cufflinks Assembly & DE v2.0 App to create DIFF tab-delimited files containing differential gene expression testing between controls and cases. We used pairwise differential expression analyses to determine differentially expressed genes. We used cuffquant/cufflinks to perform fragment bias and multi-read corrections and cuffquant/cufflinks/cuffdiff to correct for read length. Parameters were set to detect microRNAs. We used Cuffdiff to calculate the log_2_ (Ratio) statistic (Log2 fold change of the comparison over control groups) as log2 (FPKM_DCM_cases/FPKM_controls). The test statistics T was computed as E[log(y)]/Var[log(y)] where y = ratio of the normalized counts (FPKM_DCM_cases/ FPKM_controls) between controls and DCM cases. Cuffdiff used a t-test to compute the probability value (*p*-value) for genes that were differentially expressed. We reported the q-value (q) ((FDR (false discovery rate) adjusted p-value) for differential expression (a significance filter). We classified the significance statistic as “True” if the q-value (q < 0.05) was less than the false discovery rate (default: 0.05). (basespace.illumina.com).

#### Missing data

We defined data that were missing according to Cufflinks as “LOWDATA” (having inadequate sequenced reads), “HIDATA” (having numerous fragments in a locus) and “FAIL” (if the software was not able to successfully test the data). We reported data that were classified as “OK” signifying that the test was successful (basespace.illumina.com).

#### RNA-Seq variant annotation

W*e* used VCF files in the EDGC (Eone-Dianomics Genome Center) Annotator App (version 1.0. EDGC) to annotate the called RNA-Seq variations.

#### Exome sequence variant analyses

We uploaded data from the NCBI SRA website to BaseSpace using the SRA (*Sequence Read Archive)* Import App. We used the Fastq Toolkit App to filter the data for quality and read length. We set the target Phred quality score at 30, which is standard. We set the minimum read length at 32 bp (the default value). We aligned reads to the human genome (hg19), and variants were called using the BWA or Isaac Enrichment software. The EDGC (Eone-Dianomics Genome Center) Annotator App, version 1.0 was used to annotate the data.

### Pathway analyses

#### Canonical pathway analyses

We uploaded genes that were differentially expressed and statistically significant at a q < 0.05, to Ingenuity Pathway Analysis (IPA) to identify functional relationships. We used the Fisher’s exact test to discover relationships between sets of genes, pathways, and functions. (Ingenuity Systems, http://www.ingenuity.com)

#### Network analysis

We used core analyses to uncover direct and indirect interactions between putative modifiers and mutations in the network and applied p-scores to rank networks. (Ingenuity Systems, http://www.ingenuity.com)

#### Diseases and functions

We used these IPA analyses to discover diseases and functions in which genes that are differentially expressed in our data set played a role. We used the *p*-value (p) to assessment the likelihood that the association between a set of genes in our data set and a process is due to random chance. We set the alpha level of significant pathways at *p* < 0.05, indicating non-random association. IPA uses the right-tailed Fisher’s Exact test to is compute the p-value. (Ingenuity Systems, http://www.ingenuity.com).

### Data integration

**Fig. 8:**
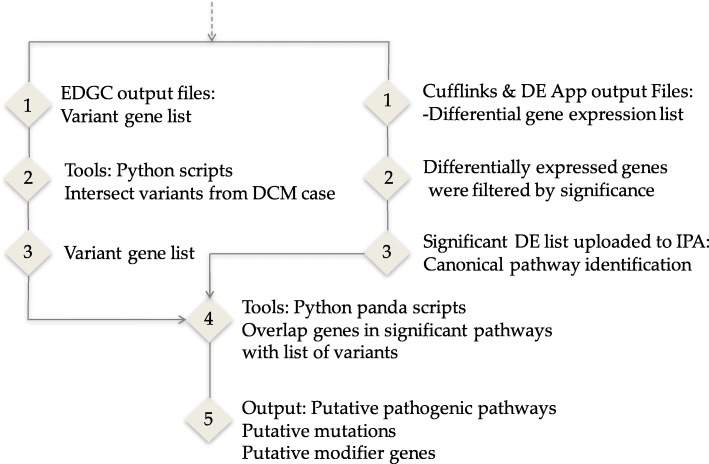
Pathway and “omics” data integration

We created a list of differentially expressed genes (q < 0.05) between drDCM cases and controls and used it to discover pathways related to our experimental data set (Fig. 8).

We overlapped the variant lists for the six drDCM patients and used the intersection to generate one list with variants discovered in all cases. To integrate the “omics” data and pathways, we overlapped genes found in significant pathways with the variant list found at the intersection of all cases (Fig. 8).

We stratified variants stratified by genotype and physically examined the quality at the respective loci. We inspected variants that passed the genotype quality filters in all drDCM cases and controls and considered variants that were still found in all cases for verification in the exome sequence data sets and ranked for reproduction analyses. We examined drDCM variations in the data sets of cases with DCM (DCM pedigrees), the Hap Map Project, and one Italian control. Variants that were found in some members of at least one DCM pedigree and not found in controls were deemed to be possible putative mutations. If they were found in controls as well, they were considered potential putative effect modifiers and prioritized for the replication analyses.

### Replication analyses

We performed association analyses to confirm findings in the drDCM data set. We assumed that a subset of patients with DCM would have drDCM. We performed chi-square tests using R-Studio (version 0.99.473–2009-2015) to determine whether variants were independent of drDCM or associated with each other. We identified associations among variations as interactions. For these analyses, we set the alpha level at < 0.05. We defined DNA changes with a direct association with drDCM (considering the subject’s genotype) as putative mutations. We described variants as putative effect modifiers if their interaction with other variations was associated with drDCM.

### Pathway and gene prioritization

We based the putative effect modifier and pathogenic pathway prioritization criteria on how connected the putative mutations were to other pathways. We presumed that the more pathways a putative mutation contributed to, the more important the influence it would have on the disease pathology. A gene contributing to several pathways was regarded a hub and selected for disease modeling. Pathways associated with our data set carrying at least one hub were considered alleged pathogenic pathways and selected for further evaluations.

## Results

### RNA-Seq drDCM data quality

The total number of reads generated from each sample ranged from 43,647,720 to 75,213,034 with a mean of 58,884,453 across all samples. The average total number of reads passing filter for controls was 65,016,348 and for cases, 54,796,523. We have presented the percentage and number of reads mapped before, and after filtering for each subject in Additional file 1: Table S2 Data produced an average value of 58,884,453 reads per sample, which meets the criteria for sequence coverage for transcriptome profiling [21].

#### Exome re-sequence data coverage

We have presented coverage data for exome sequence data sets for the Italian (SRA Study # SRP022855) (Additional file 1: Table S3) and Chinese (SRA Study # SRP066837) families in Additional file 1: Table S4. We have also given coverage data from the Hap Map project for the Asian population (SRA Study #SRP004364), and the Caucasian population (SRA Study # SRP004078) in Additional file 1: Table S5.

### Transcriptome profiling

**Fig. 9:**
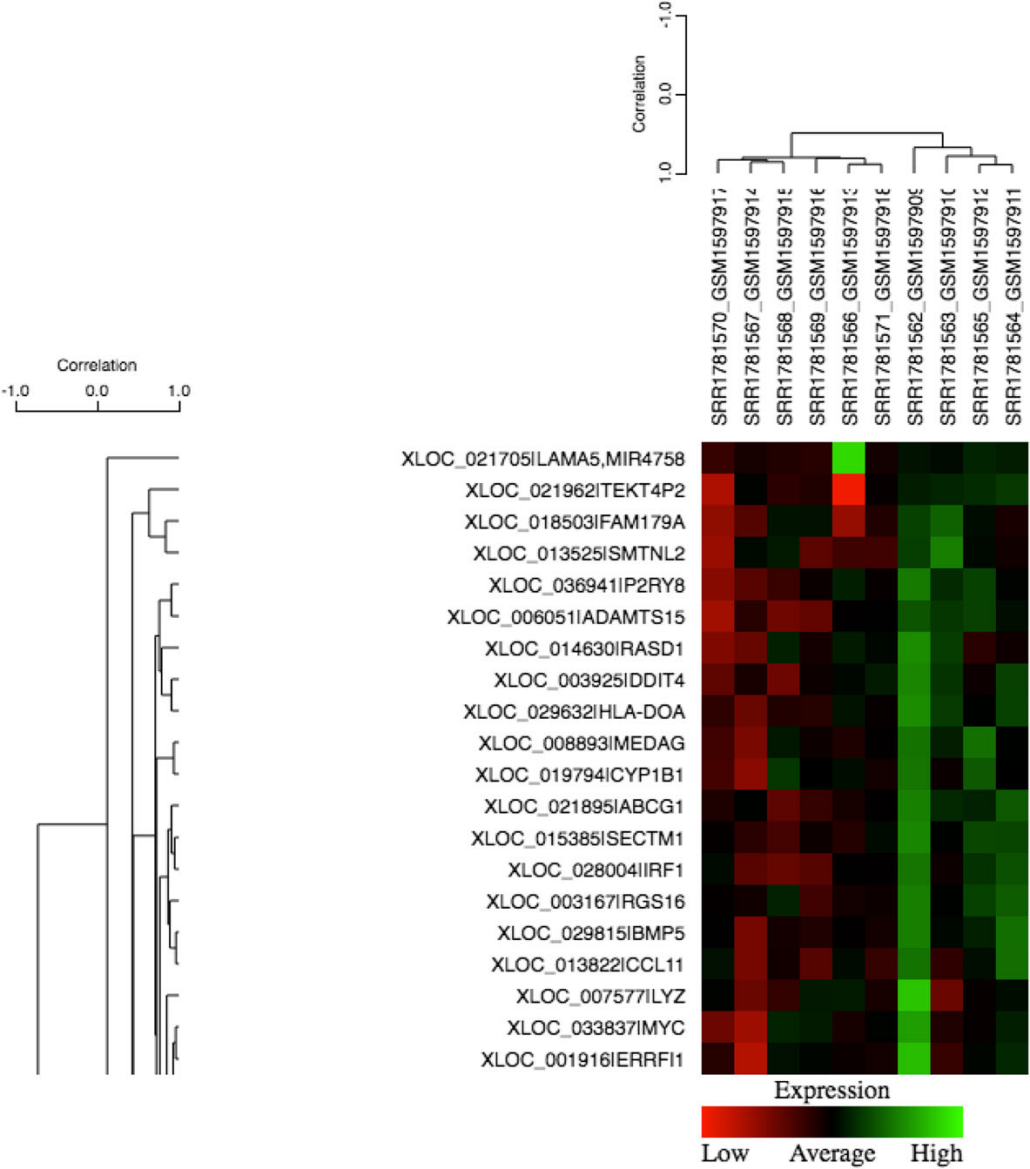
Heat map: drDCM cases vs. controls transcriptome profiling

There were 85,897 novel and known transcripts in the annotation. Of these, the annotation gene count was 37,744, of which 19, 096 genes were present for analyses. Of the 19, 096 genes, 3215 had a statistically significant differential expression (q < 0.05). We have presented a heat map to profile the expressed genes in drDCM cases versus controls in Fig. 9. The heat map indicates that in general there were more genes down-regulated in the drDCM patients compared to the controls.

### RNA-Seq drDCM variant list

**Fig. 10:**
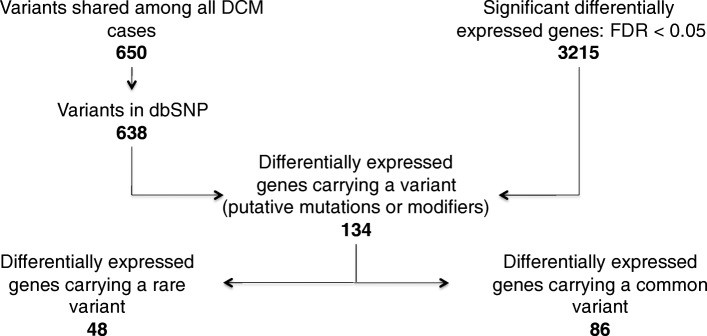
Gene and variant filtration work flow

The number of variants each patient carried ranged from 6506 to 15,717 on average; each patient carried 13,251 variations. The variant list created from the intersection of the six drDCM cases contained 650 variants (Fig. 10), of which 638 were found in dbSNP. Data revealed 134 (131 shown) differentially expressed genes carrying at least one variant, of which 48 variants were rare (Additional file 1: Table S6).

### Data integration

**Fig. 11:**
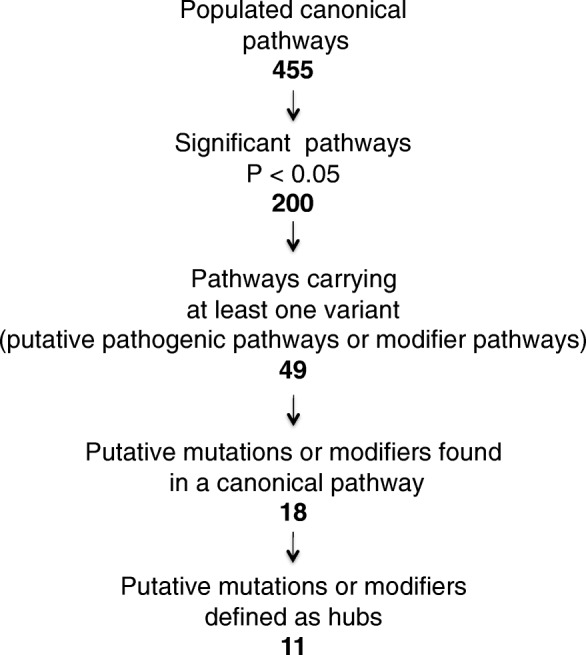
Pathway, Transcriptome, and Variant Integration

IPA generated 455 pathways of which 200 were statistically significant and associated with the drDCM data set (Additional file 1: Table S7). We overlapped the 650 variants found in all drDCM with the significant differentially expressed genes located in the 200 overrepresented pathways (Fig. 11).

Data showed that 49 (46 shown) differentially expressed genes carried at least one possible putative mutation or effect modifier. In all, there were 18 potential putative mutations or effect modifiers found in a possible putative pathogenic pathway or effect modifier pathway (Additional file 1: Table S8).

### Pathway and gene prioritization

Using the gene prioritization filter, we classified eleven genes as hubs and in this paper, we report results for the ECHS1 (enoyl-CoA hydratase, short chain, 1, mitochondrial), also known as SCEH and ECHS1D) [https://www.ncbi.nlm.nih.gov/gene/1892]. This hub mediates cross-talk among three pathways associated with the mitochondria.

### Variants associated with BCAAs and LVLFAs

Results show that there is an association between ECHS1, DBT, and MCCC1 with branched-chain amino acid and fatty acid catabolism (Table 1).

**Table 1:** Variants in the mitochondria associated with drDCM

Gene_Chr: POS	rs #	AA Change	log2 (fold change)	*p*-value	q-value	MAF/ MinorAlleleCount	Functional Consequence
ECHS1_chr10: 135186806	rs10466126 GTC ⇒ GCC	11 V/A V [Val] ⇒ A[Ala]	1.63329	0.00135	0.013927	A = 0.2448 (ExAC), A = 0.3241 (1000 Genomes), A = 0.3952 (GO-ESP)	missense, upstream variant 2 KB
ECHS1: chr10: 135184126	rs1049951 ACC ⇒ ATC	75 T/I T [Thr] ⇒ I [Ile]	1.63329	0.00135	0.013927	G = 0.0869/10314 (ExAC), G = 0.1787/895 (1000 Genomes), G = 0.1987/2583 (GO-ESP)	missense
DBT chr1:100672060	rs12021720 GGT ⇒ AGT	384G/S G [Gly] ⇒ S [Ser]	0.955072	0.00545	0.0368792	*T* = 0.0862/10470 (ExAC), *T* = 0.1082/542 (1000 Genomes), *T* = 0.1408/1831 (GO-ESP)	missense
MCCC1 chr3:183037421	rs2270968 CAC ⇒ CCC	329H/P H [His] ⇒ P [Pro]	1.61882	0.0001	0.00205554	*T* = 0.3752/45346 (ExAC), G = 0.4649/2328 (1000 Genomes), *T* = 0.4152/5400 (GO-ESP)	intron variant, missense, nc transcript variant

Legend: *ECHS1* enoyl-CoA hydratase, short chain, 1, mitochondrial, *DBT* Dihydrolipoamide branched chain transacylase E2, and *MCCC1* methyl crotonoyl-CoA carboxylase 1. *AA* Amino acid. *P-value* probability value. *Q-value* p-value adjusted for the false discovery rate

ECHS1 is up-regulated (log_2_ (fold change) = 1.63329) and associated with drDCM (q = 0.013927). It carries two common missense variants, V11A (*ECHS1*:rs10466126) in the first exon, and T75I (*ECHS1*:rs1049951) in the second exon (Table 1). DBT is also up-regulated (log_2_ (fold change) = 0.955072) and associated with drDCM (q = 0.0368792). It also carries a common missense variant 384G/S (*DBT*:rs12021720) (Table 1). An additional common missense variant was found in the *MCCC1*:rs2270968. This gene codes the subunit of 3-methylcrotonyl-CoA carboxylase, an enzyme that catalyzes the carboxylation of 3-methylcrotonyl-CoA to form 3-methylglutaconyl-CoA. [22] It is up-regulated (log_2_ (fold change) = 1.61882) and associated with degradation of the branched-chain amino acid, leucine. It is also associated with drDCM (q = 0.00205554) (Table 1).

### drDCM patient genotypes

Results show that all cases are homozygous for the *ECHS1*:rs10466126 and *ECHS1*:rs1049951 variants (Additional file 1: Table S9). One control is heterozygous for *ECHS1*:rs10466126. One unaffected is homozygous and another heterozygous for the *ECHS1*:rs1049951 variant. For the *DBT*:rs12021720 variant, cases are either homozygous or heterozygous, and one control is heterozygous (Additional file 1: Table S9).

#### ECHS1 variant verification in FAM001 and FAM027

After scanning the additional exome sequence data sets, results show that there are no patients from FAM001 who are recessive for the *ECHS1*:rs10466126 variant. Three out of four patients from FAM027 are heterozygous. However, two out of the five Chinese patients are homozygous (G/G). All patients in families FAM001 and FAM027 who carried the *ECHS1*:rs1049951 SNP are homozygous. Two of the three who carried the *ECHS1*:rs1049951 variant are heterozygous, and one is homozygous in the Chinese family. Two patients in the Chinese family did not have the SNP (Additional file 1: Table S10).

#### Variant verification in FAM001, FAM027, and Chinese

Cases in all families are homozygous for the 384S/G (*DBT*:rs12021720) variant apart from one Chinese member (SRR2968053) who do not carry the variant (Additional file 1: Table S10). All cases apart from one in FAM001 and 027 are either homozygous or heterozygous for the *MCCC1*:rs2270968 variant. We did not find this variant in family members from China (Additional file 1: Table S10).

#### Variant verification in the hap map project

Results from scanning subjects from the Hap Map project (with unknown phenotype) and Italy (healthy control) show that no subject in this population carries the *ECHS1*:rs10466126 (G/G) recessive genotype. The three Caucasian subjects used to match Fam027 are heterozygous (A/G). However, it is necessary for one to note that the data had insufficient quality for these three individuals. Subjects are either recessive or heterozygous for the other variants (Additional file 1: Table S11). From these results, we determined that the *ECHS1*:rs10466126 (G/G genotype) variant is a possible putative mutation. It is found in some other patients with familial DCM and is found only in cases and no controls.

### *ECHS1*:rs10466126 **population genetics**

We used Ensemble (1000 Genome Project Phase 3 [23]) to evaluate the frequency of the *ECHS1*:rs10466126 (G/G) variant in different world populations. Results reveal that the allele frequencies vary widely. For example, the frequency in the 1000 genome project is A: 0.324 (count = 1623) and G: 0.676 (count = 3385). However, in the African population the allele frequency for the A: 0.717 (count = 948) and G: 0.283 (count = 373) is very different. In the Han Chinese in Beijing, China population it is A: 0.058 (count = 12) and G: 0.942 (count = 194). In the British in England and Scotland it is A: 0.236 (count = 43) G: 0.764 (count = 139). We have presented population allele frequencies from the additional 1000 Genome project populations in Additional file 1: Table S12.

### Novel variant in the ECHS1 gene

**Fig. 12:**
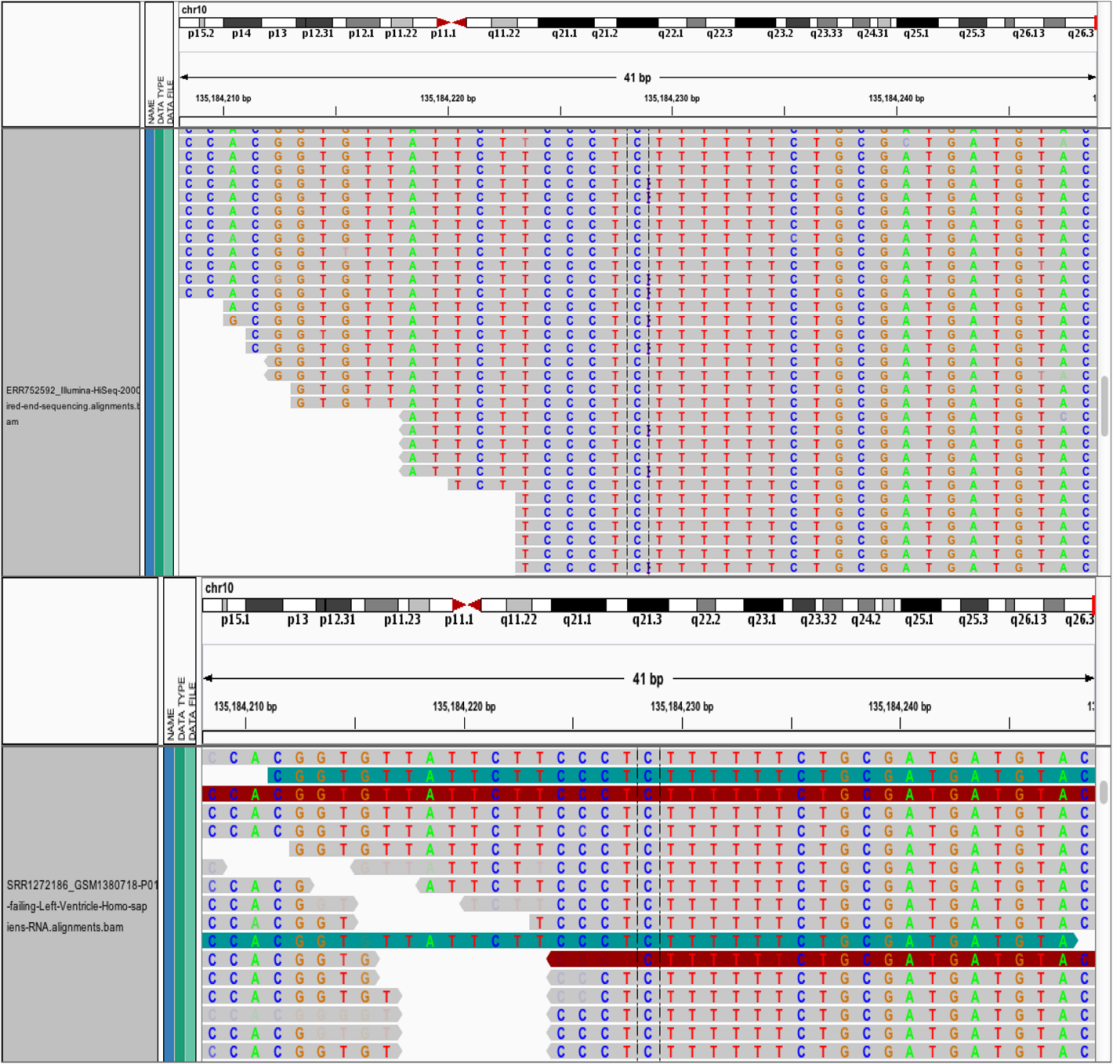
Novel ECHS1 c.41insT

We scanned the ECHS1 gene for novel variants after observing that apart from carrying two variants, one in the first exon and the other in the second exon, *ECHS1* also hosts a microRNA, MIR3944 in the first intron. After scanning *ECHS1* for novel variants, we found an indel, *ECHS1*:c.41insT that causes a frameshift and elongation on chromosome 10, position 135,184,228, of the second exon (Fig. 12). This variant is found in five of the six drDCM cases and is absent in all controls (Additional file 1: Table S13).

### Evaluation of *ECHS1*:rs10466126 and *ECHS1:*rs1049951

The *ECHS1*:rs10466126 is found in exon 1 and *ECHS1:* rs1049951 in exon 2. These variants are both missense variants. *ECHS1*:rs10466126 changes the amino acid from Valine to Alanine and *ECHS1:* rs1049951 from Threonine to Isoleucine. Both variants are common in the general population (Table 1). We used the Ensembl Variant Effect Predictor (connected to UCSC database hg38) at <https://genome.ucsc.edu/cgi-bin/hgVai> to evaluate the effect of the variants on the gene at the protein level. Results show that as exonic variants, both are tolerated, SIFT = T (0.494) for *ECHS1*:rs10466126, and SIFT = T (0.149) for *ECHS1:* rs1049951.

**Fig. 13:**
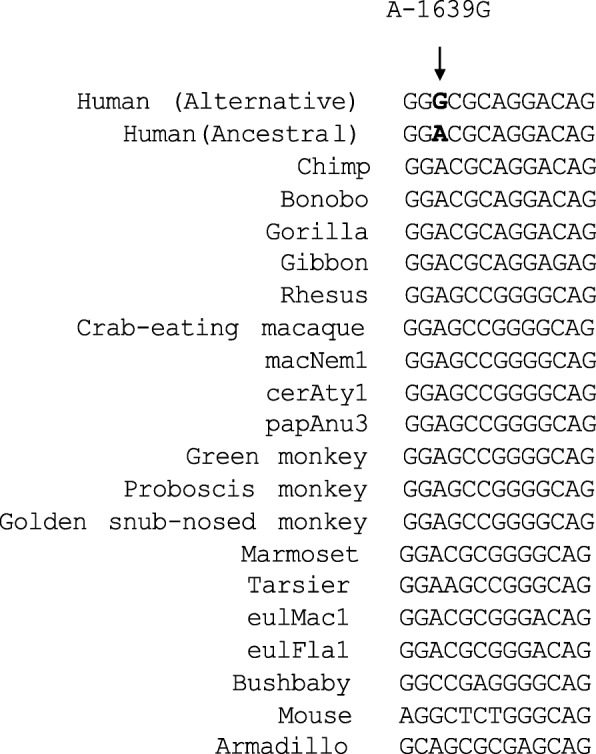
Conservation of A-1639G (*ECHS1*:rs10466126 (A/A)) among species

The next step of the analyses was to determine whether the *ECHS1*:rs10466126 (G/G) variant is conserved among species and if it is found in a region of the gene where it would interfere with gene regulation. We used Multiz in UCSC genome browser comparative genomics alignment pipeline at < genome.ucsc.edu > to generate the multiple comparison alignments and determine which *ECHS1*:rs10466126 allele is conserved among species. Results show that the ancestral allele (A) is conserved (Fig. 13).

**Fig. 14:**
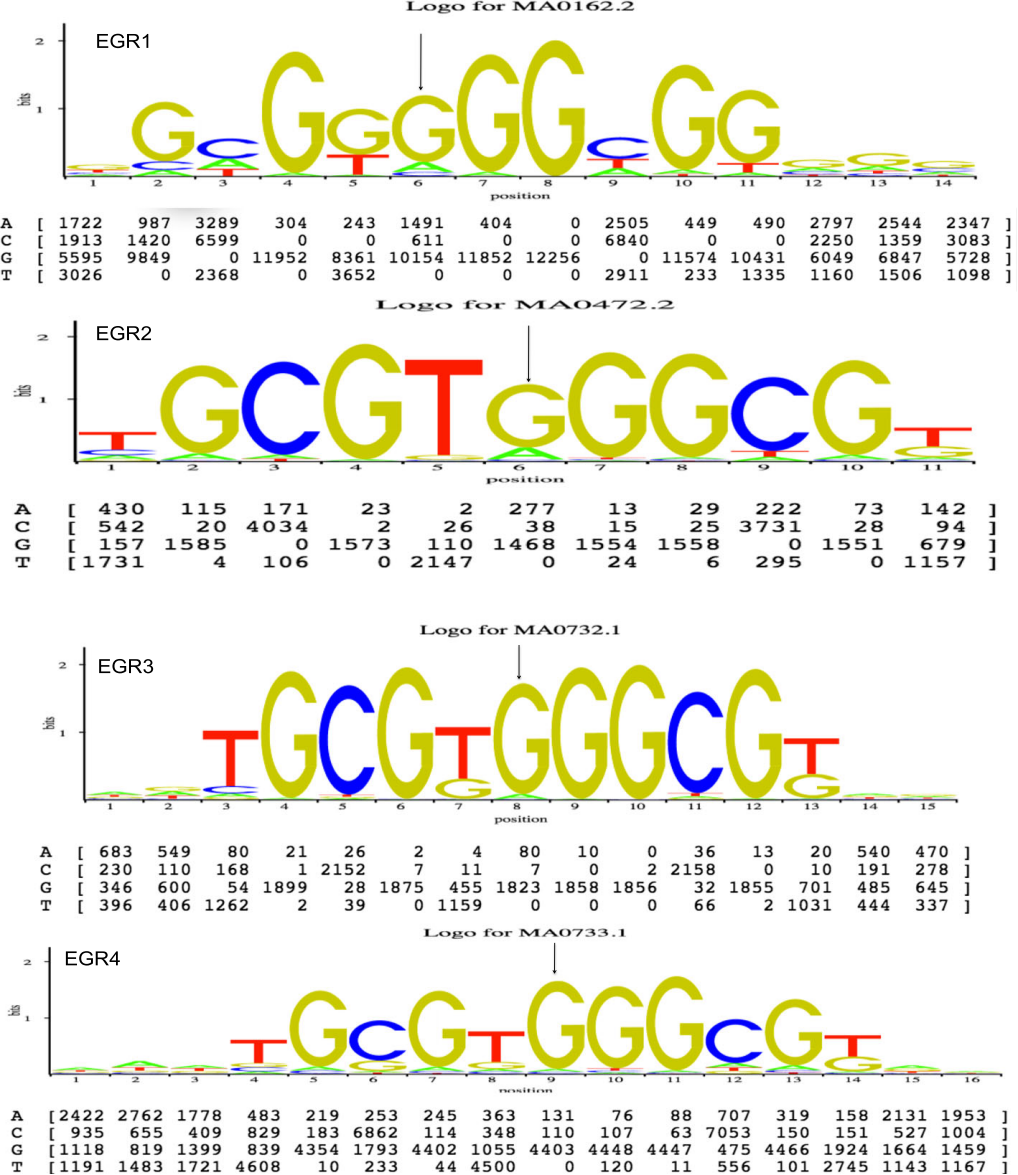
JASPAR prediction analysis. Legend: Figure shows the 5’-GCG(T/G)GGGCG-3′(EGR-site DNA sequence) and the ECHS1:rs10466126 (G/G) locus. The arrow indicates the ECHS1:rs10466126 (G/G) locus. Shown in these figures are a sequence logo, a count matrix, and hits/bp statistics. It is a graphical representation of the matrix model, based on the information content. The information content of a matrix column ranges from 0 (no base preference) and 2 (only one base used). The sequence logo is a barplot showing the total information content at each position. Bars are replaced by stacked letters (A,C,G,T), which are sized and sorted relative to their occurrence. The count matrix is the underlying model showing the DNA pattern. The numbers in cells indicate the number of sequences having base x in column y [23]

We used the JASPAR database [24] to evaluate *ECHS1*:rs10466126 putative mutation as an upstream gene variant and whether it effects the gene’s regulation. We wanted to assess whether *ECHS1*:rs10466126 (G/G) is in a regulatory region and if so what transcription factors bind to the locus. The analyses revealed that the *ECHS1*:rs10466126 (G/G) variant is located in a regulatory motif 5’-GCG(T/G)**G**GGCG-3′(EGR-site DNA sequence) and that four EGR (early growth response protein 1, 2, 3 and 4) transcription regulators bind to it (Fig. 14).

Interestingly, data showed that the EGR transcription factors all bind to the G allele (EGR1 frequency: 10154, EGR2 frequency: 1468, EGR3 frequency: 1823 and EGR4 frequency: 4403). The binding of EGR transcription factors to the alternative G allele occurs more often than to the ancestral A allele (EGR1 frequency: 1491, EGR2 frequency: 277, EGR3 frequency: 80, and EGR4 frequency: 131) (Fig. 14). Results suggest that *ECHS1*:V11A (rs10466126) is in a regulatory motif of four EGR (early growth response protein 1, 2, 3 and 4)-transcription regulators.

To establish consistency, we used the University of California, Santa Cruz (UCSC) genome browser at https://genome.ucsc.edu/index.html to determine what transcription factors bind to ECHS1. Results showed that several transcription factors bind to ECHS1. Further inquiry of regulators that attach to the first exon from UCSC revealed three transcription factors, MITF (melanogenesis associated transcription factor), ETS1 (ETS proto-oncogene 1, transcription factor) including EGR1 (Additional file 2:Figure S1).

After finding that the EGRs transcription regulators bind to the *ECHS1*:rs10466126 (G/G) locus located in the 5’-GCG(T/G)**G**GGCG-3′(EGR-site) DNA sequence, we scanned the transcriptome data set and found that EGR1 (q-value = 0.0281271; log_2_ (fold change) = − 1.29395), EGR2 (q-value = 0.0494839; log_2_ (fold change) = − 1.23546), and EGR3 (q-value = 0.00891741; log_2_ (fold change) = − 2.06029) transcription factors were all down-regulated in cases versus controls (Additional file 1: Table S14). We also found that EGR1 and EGR3 transcription factors were significantly associated with cardiovascular system development and function, development of endothelial tissue (*p*-value = 7.43E-12; z-score = − 3.140) (Additional file 1: Table S15). This function is also decreased in drDCM. Together, these findings suggest that *ECHS1*:rs10466126 (G/G) might be adversely affecting gene regulation associated with EGR1 through the regulation of transcription. Thus, even though both exonic variants are tolerated, the putative mutation, *ECHS1*:rs10466126 (G/G) also found upstream of the gene lies in a regulatory region and appears to affect gene regulation.

### ECHS1 is a host for miRNA3944

**Fig. 15:**
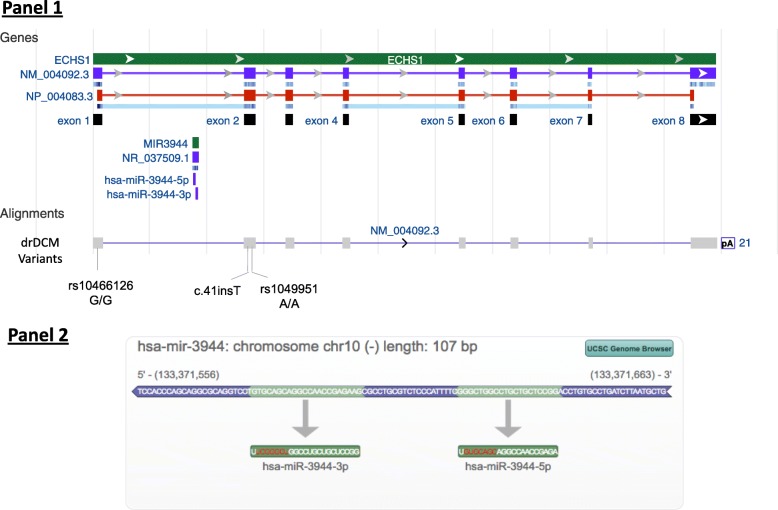
Schematic of ECHS1 and MIR3944 using miRIAD. Legend: Panel 1: Summarized transcript annotation indicating the exons, introns, position of the MIR3944, and the variants examined in the study. Panel 2: Schematic of has-mir-3944 indicating the seed sites for the guide, has-miR-3944-3p and passenger strand, has-miR-3944-5p microRNAs. Highlighted in red are the seed sites

*ECHS1* is found on chromosome 10. It has eight exons and has seven introns (Fig. 15, Panel 1). In the previous section, data revealed a “hot spot” in *ECHS1* spanning the first exon, intron, and the second exon. Data showed that the *ECHS1:*rs10466126 is in an EGR regulatory motif of the first exon. The other two variants, the *ECHS1:*rs1049951, and *ECHS1*:c.41insT are both in the second exon. Interestingly in the first intron, the gene hosts a mirtron, the MIR3944 (Fig. 15, Panel 1).

Investigations into the nature of MIR3944 using miRIAD, (an intragenic miRNA database) (https://www.bioinfo.mochsl.org.br/?q=tools) revealed that the precursor sequence of MIR3944 has a functional passenger strand, the has-miR-3944-5p and a functional guide strand, the has-miR-3944-3p. The length of has-miR-3944 is 107 bp. Color-coded in red is the seed sites for has-miR-3944-3p and has-miR-3944-5p (Fig. 15, Panel 2).

The miRIAD database also revealed that ECHS1 is expressed in the healthy heart (Additional file 3: Figure S2, Panel 1). However, MIR3944 is not expressed in the healthy heart (Additional file 3: Figure S2, Panel 2). Additionally, the miRIAD database revealed expression correlations between has-mir-3944-5p and the host gene. Data show that when ECHS1 is expressed in the heart (green dot), has-mir-3944-5p is not (Additional file 3: Figure S2, Panel 3).

**Fig. 16:**
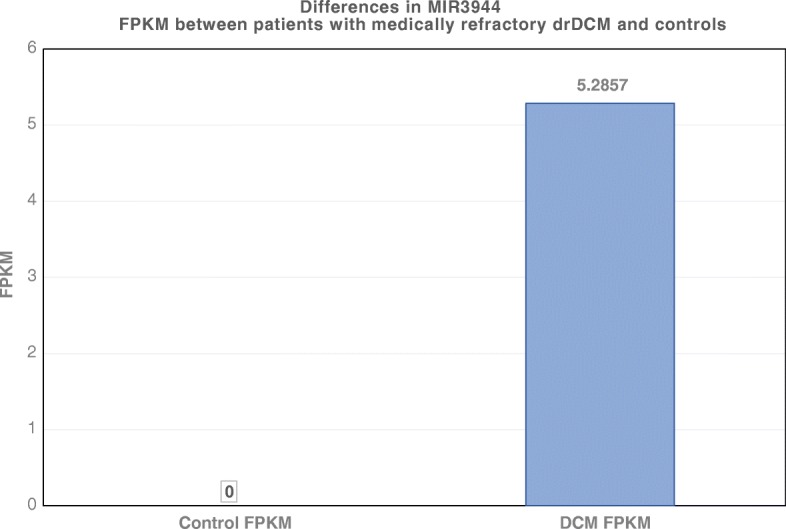
Expression profiles of MIR3944 in drDCM

In this study, data show that MIR3944 is expressed in patients with drDCM (case FPKM = 5.2944) but not in the controls (FPKM = 0) (Fig. 16).

#### RHOD is a putative target for has-miR-3944-5p

**Fig. 17:**
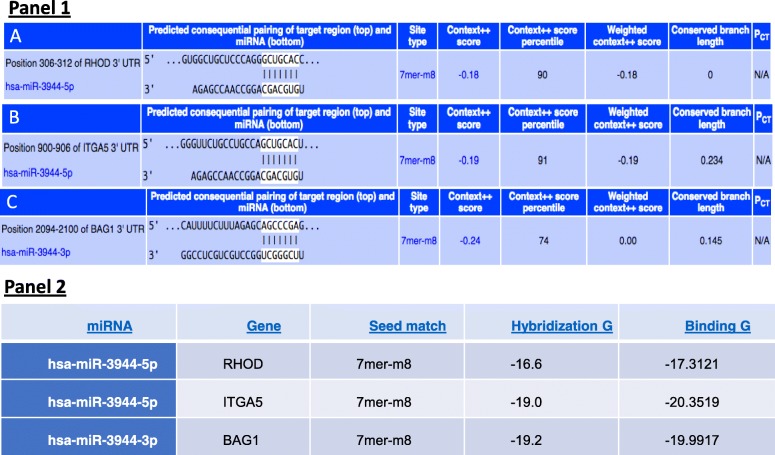
TargetScan predicted seed sites pairing with target region. Legend: Panel 1: Predicted consequential pairing of the target region and mircoRNA. Panel 2: The table shows gene targets predicted by seed match (8mer,7mer-m8,7mer-1A) according to TargetScan 6.2 http://bmi.ana.med.uni-muenchen.de/miriad/miRNA/human/hsa-mir-3944/

We used TargetScanHuman: prediction of microRNA targets (release 7.1: June 16) [25] to determine what genes hsa-mir-3944 might target. TargetScanHuman predicted that hsa-mir-3944-5p binds to position 301–312 of RHOD 3’UTR. The projected consequential pairing of the target region and the seed site in the miRNA is 100% (Fig. 17, Panel 1A). The table in Fig. 17, Panel 2 shows putative gene targets predicted by seed match (8mer,7mer-m8,7mer-1A) according to TargetScan 6.2. RHOD is a 7mer-m8, suggesting that there is an exact match to positions 2–8 of the mature microRNA (i.e., the seed site plus the 8th position) (http://www.targetscan.org/docs/7mer.html).

#### ITGAV is a putative target for has-miR-3944-5p

TargetScanHuman also predicted that has-miR-3944-5p (seed site (GUGCAGC) bids position 900–906 of ITGAV 3’UTR (Fig. 17 Panel 1B). There is 100% predicted consequential pairing of the target region and the miRNA seed site (Fig. 17 Panel 1B). The Table in Fig. 17, Panel 2 shows putative gene targets predicted by seed match (8mer,7mer-m8,7mer-1A) according to TargetScan 6.2. ITGAV is a 7mer-m8, suggesting that there is an exact match to positions 2–8 of the mature microRNA (i.e., the seed site plus the 8th position) (http://www.targetscan.org/docs/7mer.html).

#### BAG1 is a putative target for has-miR-3944-3p

TargetScanHuman also predicted that the guide strand, has-miR-3944-3p (seed site sequence (UCGGGCU) bids position 2094–2100 of BAG1 3’UTR with 100% predicted consequential pairing with the miRNA seed site (Fig. 17 Panel 1C). The Table in Fig. 17, Panel 2 shows putative gene targets predicted by seed match (8mer,7mer-m8,7mer-1A) according to TargetScan 6.2. BAG1 is also a 7mer-m8, suggesting that there is an exact match to positions 2–8 of the mature microRNA (i.e., the seed site plus the 8th position) (http://www.targetscan.org/docs/7mer.html).

Prediction analyses for RHOD indicate that only a segment (UGCA) of the target site is conserved among species (Additional file 4: Figure S3A). For ITGAV, the target site appears to be conserved among species (Additional file 4: Figure S3B). The BAG1 target site is conserved among human, Chimp, and Rhesus (Additional file 4: Figure 3c).

The next step in modeling drDCM was to determine which variants were putative mutations or effect modifiers. To do this, we replicated the variant detection analyses in a more extensive and independent sample of patients with DCM and subjects without DCM.

### Variant detection replication analyses

Association analyses showed that there is dependency of the *ECHS1*:rs10466126 variant (considering genotype) on DCM (χ-squared = 7.7434, *p* = 0.02082). After finding this association, we stratified by genotype and found that the *ECHS1*:rs10466126 (G/G) recessive genotype is associated with DCM (*p* = 0.02496) and not the *ECHS1:*rs10466126 (A/G) heterozygote genotype (*p* = 0.209) (Table 2). Results suggest that *ECHS1*:rs10466126 (G/G) is the putative mutation when one considers the genotype of the subjects.

**Table 2:** Variant detection replication analyses

	Pearson’s Chi-squared tests with Yates’ continuity correction: *p*-value	X-squared	df
ECHS1 main effects			
Exon 1: rs10466126 and Exon 2: rs1049951			
Exon 1: rs10466126 SNP and disease state	0.1718	1.8674	1
Exon 2: rs1049951 SNP and disease state	* < 2.2e-16	88.338	1
Exon 1:rs10466126 (genotype level) and disease state	0.02082	7.7434	2
Exon 2: rs1049951 (genotype) and disease state	1	0	1
*rs10466126 (G/G) and disease state	0.02496	5.0269	1
rs10466126 (A/G) and disease state	0.209	1.5782	1
Interactions			
Exon 1: rs10466126 SNP and Exon 2: rs1049951 SNP	* < 2.2e-16	101.4085	1
*Exon 2: rs1049951 (genotype) and Exon 1: rs10466126 (genotype)	*2.82E-07	30.1636	7
*rs10466126 (genotype), rs1049951 (genotype) and disease state	* < 2.2e-16	382.4507	11
Exon 2: ECHS1 c.41insT main effects			
Exon 2: ECHS1 c.41insT (genotype) and disease state	0.3218	0.9816	1
Interactions			
Exon 2: rs1049951, Exon 1: rs10466126 and Exon 2: ECHS1 c.41insT	* < 2.2e-16	147.8592	3
Exon 1: rs10466126 and Exon 2: ECHS1 c.41insT	0.714	0.1343	1
*Exon 2: rs1049951 and Exon 2: ECHS1 c.41insT	*7.84E-08	28.8451	1
*Exon 2: rs1049951 and Exon 2: ECHS1 c.41insT and disease state	* < 2.2e-16	129.6056	3
DBT main effects			
rs12021720 and DCM	1	0	1
rs12021720 (genotype) and DCM	*0.3232	2.2589	2
Interactions			
DBT rs12021720 and ECHS1 rs10466126	0.005657	7.6566	1
*DBT rs12021720 (genotype) and ECHS1 rs10466126 (genotype)	*0.001096	18.2641	4
DBT rs12021720, ECHS1 rs10466126, and DCM	* < 2.2e-16	653.831	7
*DBT rs12021720 (genotype) and ECHS1 rs10466126 (genotype) and DCM	* < 2.2e-16	427.9155	17

Legend: *Indicates Chi-squared test for probabilities *p*-values

Interestingly, when we analyzed the association of *ECHS1*:rs1049951 with DCM, results showed that rs1049951 is highly dependent on DCM (*p* < 2.2e-16). However, when we took subject genotype into consideration, there was no longer an association and results showed that *ECHS1:*rs1049951 is independent of DCM (*p* = 1). However, there is an association between *ECHS1*: rs1049951 (genotype) and *ECHS1*:rs10466126 (genotype) (*p* = 2.82E-07) and this interaction is associated with DCM (p < 2.2e-16) (Table 2).

### Linkage disequilibrium evaluation of *ECHS1*:rs10466126 and *ECHS1:*rs1049951

After finding that there is a statistically significant association between *ECHS1*:rs10466126 and *ECHS1:*rs1049951 we used Ensembl to perform pairwise linkage disequilibrium analyses to determine whether these two variants are independent or co-inherited. Results show that these two variants are in strong linkage disequilibrium in the Han Chinese in Beijing, China population (1000 Genomes: phase_3: CHB) (D’ = 1). This population matches the ethnicity of our drDCM Chinese dataset. Additional linkage disequilibrium evaluations of *ECHS1*:rs10466126 and *ECHS1:*rs1049951 in other populations can be found in Additional file 1: Table S16.

#### Replication analyses for ECHS1:C.41insT

Data show that 39 subjects out of 143 carried the *ECHS1*:c.41insT genotype, two of whom were controls (frequency in case = 0.258741, and in study population = 0.272727). In our study population, the frequency of *ECHS1*:c.41insT is 0.273, and the chi-square test showed that the *ECHS1*:c.41insT(CT) genotype is independent of DCM (*p* = 0.3218). However, there is significant dependence or interaction between the *ECHS1*:rs1049951 and the *ECHS1*:c.41insT variant (*p* = 7.841e-08), which are both in the second exon. Additionally, there is an association between this interaction and disease state (p < 2.2e-16). Because there is no direct relationship between *ECHS1*:c.41insT and disease state, we defined it as a putative effect modifier (Table 2).

#### Replication analyses for DBT

Results from the chi-square test showed that *DBT*:rs12021720 is not directly related to DCM (*p* = 0.3232). However, interaction analyses showed that there is an association between *DBT*:rs12021720 (genotypes) and *ECHS1*:rs10466126 (genotypes) (*p* = 0.001096). We defined DBT as a putative effect modifier.

Results show that there is an association between *ECHS1*:rs10466126 (G/G) genotype and drDCM and that it is a putative mutation, as data showed in the primary study. Collectively, these results suggest that individuals carrying the *ECHS1*:rs10466126 (G/G) genotype have a high chance of developing drDCM. Those who carry both *ECHS1*:rs10466126 (G/G) and the *ECHS1*:rs1049951 SNP and *ECHS1*:c.41insT are double and triple mutants, respectively, and have an even higher chance of developing drDCM. The risk of developing drDCM could even be more significant in patients who also carry the *DBT*:rs12021720 variant. The next step was to determine the putative pathogenic pathways associated with ECHS1.

### Putative pathogenic pathways related to ECHS1

Results show that three different metabolic pathways carry ECHS1 and DBT, the valine (−log (p) = 3.39) and isoleucine degradation 1 pathways (−log (p) = 2.34), and the fatty acid β-oxidation pathways (−log (p) = 2.83), which are all associated with the drDCM data set (Table 3). Because these pathways carry the ECHS1 gene, we defined them as putative pathogenic pathways.

**Table 3:** Putative pathways carrying the ECHS1 and DBT

Canonical Pathways	DCM pathway: -log(p-value)	Molecules in DCM pathways
Isoleucine Degradation I	2.34	HSD17B10, AUH, DLD, ACADSB, BCAT2, ACAT1, HADHA, EHHADH, ECHS1, DBT
Fatty Acid β-oxidation I	2.83	ACAA2, HSD17B10, HADHA, EHHADH, ECI1, AUH, ACSL5, SLC27A3, HSD17B8, ECI2, IVD, ECHS1, SLC27A6
Valine Degradation I	3.39	HIBCH, BCKDHA, AUH, DLD, ACADSB, ALDH6A1, BCAT2, BCKDHB, HADHA, EHHADH, ABAT, ECHS1, DBT

Enzymes and products generated in the valine, and isoleucine degradation 1 pathways and the fatty acid β-oxidation pathway are similar. The pathways all produce *NADH* (nicotinamide adenine dinucleotide) and H^+^ (proton). Fatty acid β-oxidation (Additional file 5: Figure S4.1) and isoleucine degradation 1 pathways (Additional file 5: Figure S4.2) also generate acetyl-CoA (*Acetyl* coenzyme A). Additionally, both the valine (Additional file 5: Figure S4.3) and isoleucine degradation 1 pathways also produce propionyl coenzyme A (propionyl-CoA). In addition to the production of propionyl-CoA, the valine degradation pathway also creates 2-oxoglutarate, and (S)-3-amino-2-methylpropanoate. Results also show upregulation of critical enzymes in the degradation processes of the two amino acid pathways in drDCM cases compared to controls (Table 4).

**Table 4:** Genes found in the isoleucine degradation 1 pathways

	Isoleucine Degradation 1 Pathways		
Symbol	Entrez Gene Name	log_2_ (fold Change)	Type(s)
ACADSB	acyl-CoA dehydrogenase, short/branched chain	1.615	enzyme
ACAT1	acetyl-CoA acetyltransferase 1	1.755	enzyme
AUH	AU RNA binding protein/enoyl-CoA hydratase	1.373	enzyme
BCAT2	branched chain amino acid transaminase 2	1.417	enzyme
DBT	dihydrolipoamide branched chain transacylase E2	0.955	enzyme
DLD	dihydrolipoamide dehydrogenase	1.287	enzyme
ECHS1	enoyl-CoA hydratase, short chain, 1, mitochondrial	1.633	enzyme
EHHADH	enoyl-CoA, hydratase/3-hydroxyacyl CoA dehydrogenase	1.776	enzyme
HADHA	hydroxyacyl-CoA dehydrogenase/3-ketoacyl-CoA thiolase/enoyl-CoA hydratase (trifunctional protein), alpha subunit	1.47	enzyme
HSD17B10	hydroxysteroid (17-beta) dehydrogenase 10	1.784	enzyme
Valine Degradation 1 Pathway			
Symbol	Entrez Gene Name	log2(fold Change)	Type(s)
ABAT	4-aminobutyrate aminotransferase	2.218	enzyme
ACADSB	acyl-CoA dehydrogenase, short/branched chain	1.615	enzyme
ALDH6A1	aldehyde dehydrogenase 6 family member A1	1.534	enzyme
AUH	AU RNA binding protein/enoyl-CoA hydratase	1.373	enzyme
BCAT2	branched chain amino acid transaminase 2	1.417	enzyme
BCKDHA	branched chain keto acid dehydrogenase E1, alpha polypeptide	1.175	enzyme
BCKDHB	branched chain keto acid dehydrogenase E1, beta polypeptide	1.252	enzyme
DBT	dihydrolipoamide branched chain transacylase E2	0.955	enzyme
DLD	dihydrolipoamide dehydrogenase	1.287	enzyme
ECHS1	enoyl-CoA hydratase, short chain, 1, mitochondrial	1.633	enzyme

Data suggests that the rate of production for NADH, H+, acetyl-CoA, propionyl-CoA, 2-oxoglutarate, and (S)-3-amino-2-methylpropanoatemay may be increased leading to an accumulation in the mitochondria of the myocardium (Additional file 6: Figure S5). In the isoleucine (Table 4) and valine degradation 1 pathways, DBT carried the *DBT*:rs12021720 variant, a putative effect modifier. This gene encodes a protein that forms the critical homo-24-meric dihydrolipoyl transacylase (E2) subunit of the branched-chain alpha-keto acid dehydrogenase complex (BCKD), an enzyme complex inside the mitochondria (https://www.ncbi.nlm.nih.gov/gene/1629).

In this study, there is an up-regulation and association of the other subunits of BCKD with drDCM. The subunits include BCKDHA (branched chain keto acid dehydrogenase E1, alpha polypeptide), log_2_ (fold change) = 1.17513; q = 0.0240843, BCKDHB (branched chain keto acid dehydrogenase E1, beta polypeptide), log_2_ (fold change) = 1.25182; q = 0.0354139, and DLD (dihydrolipoamide dehydrogenase), log_2_ (fold change) = 1.28677; q = 0.0346444 (Additional file 1: Table S14). Results indicate that the BCKD enzyme complex is up-regulated and that the rate at which both isoleucine and valine are being broken down is increased.

Interestingly, in the fatty acid β-oxidation pathway (Table 5), ACSL5 (acyl-CoA synthetase long-chain family member 5), (log_2_ (fold change) = − 1.91822; q = 0.00118756), an enzyme that activates long-chain fatty acids in the cytoplasm before transport to the mitochondria, is down-regulated.

**Table 5:** Genes in the fatty acid β-oxidation pathway

Symbol	Entrez Gene Name	log_2_(fold Change)	Type(s)
ACAA2	acetyl-CoA acyltransferase 2	1.673	enzyme
ACSL5	acyl-CoA synthetase long-chain family member 5	− 1.918	enzyme
AUH	AU RNA binding protein/enoyl-CoA hydratase	1.373	enzyme
ECHS1	enoyl-CoA hydratase, short chain, 1, mitochondrial	1.633	enzyme
ECI1	enoyl-CoA delta isomerase 1	1.282	enzyme
ECI2	enoyl-CoA delta isomerase 2	1.457	enzyme
EHHADH	enoyl-CoA, hydratase/3-hydroxyacyl CoA dehydrogenase	1.776	enzyme
HADHA	hydroxyacyl-CoA dehydrogenase/3-ketoacyl-CoA thiolase/enoyl-CoA hydratase (trifunctional protein), alpha subunit	1.470	enzyme
HSD17B8	hydroxysteroid (17-beta) dehydrogenase 8	1.475	enzyme
HSD17B10	hydroxysteroid (17-beta) dehydrogenase 10	1.784	enzyme
IVD	isovaleryl-CoA dehydrogenase	1.263	enzyme
SLC27A3	solute carrier family 27 member 3	−1.357	transporter
SLC27A6	solute carrier family 27 member 6	1.646	transporter

Results suggest that there is a reduction in the rate of activation of long-chain fatty acids. Additionally, SLC27A3 (solute carrier family 27 member 3) (log_2_ (fold change) = − 1.35713; q = 0.00347516), a very long-chain fatty acid acyl-CoA synthetase is also down-regulated. Downregulation of SLC27A3 suggests that there is a reduction in the rate of activation of very-long-chain fatty acids as well (Additional file 1: Table S14). Results indicate that there is an accumulation of long and very long chain fatty acids in the cytoplasm.

To understand the full biological implication for the significant results, we explored supporting evidence from other canonical pathways and transcripts in the data set. Interestingly, there is an association between the leucine degradation 1 pathway with the data set. We defined this pathway as a putative effect-modifying pathway because it carried the *DBT*:rs12021720 putative effect modifier.

Further pathway investigations revealed two more pathways related to catabolism of fatty acids. These included the fatty acid β-oxidation III (Unsaturated, Odd Number) (−log (p) = 1.61E00) and the fatty acid α-oxidation (−log (p) = 3.14E00) that were associated with the drDCM data set. However, these pathways did not carry the *ECHS1*:rs10466126 or *DBT*:rs12021720 variants (Additional file 1: Table S7). Data also showed that mitochondrial (−log (p) = 8.73E00), oxidative phosphorylation (−log (p) = 5.35E00) and TCA cycle II (−log (p) = 2.70E00) dysfunction were associated with the experimental data set (Additional file 1: Table S7).

### Network analyses

To gain insight into the multidimensional interactions among genes, we performed network analyses.

#### Mitochondria and cytoskeleton crosstalk

**Fig. 18:**
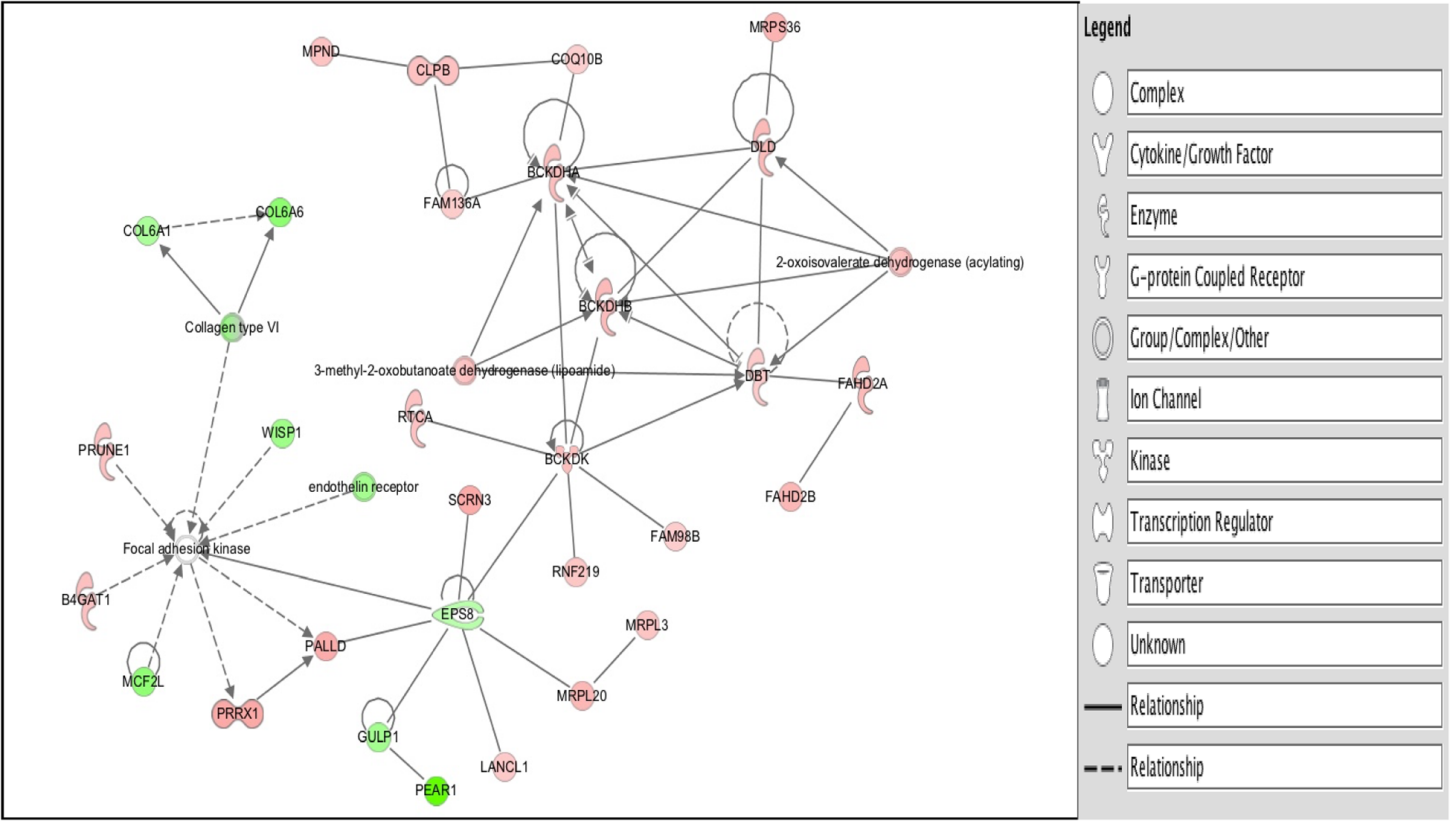
Direct and indirect network analysis. Legend: A. Cross talk between the mitochondria and the cytoskeleton. B. ITGAV directly binds to Actin. Lines indicate edges or relationships. Straight solid edges indicate binding, lines with an arrow head indicate act on, and circular line on a molecule indicate a feedback. Green molecules indicate down-regulated genes and red focus genes indicate up-regulated genes

Network analyses show that BCKDK binds to EPS8 localized in the actin filaments, and actin stress fibers of the sarcomere (Fig. 18). Additionally, EPS8 binds to PALLD (palladin, cytoskeletal associated protein) that localizes in the Z line, actin bundles, actin cytoskeleton, actin filaments, actin stress fibers, intercalated disks of the sarcomere (Additional file 1: Table S17). Results obtained suggest that there is crosstalk between the sarcomere and the mitochondria.

### Evaluations of the ECHS1 gene

We used two different databases, Harmonizome [26] and the all RNA-seq and ChIP-seq sample and signature search (ARCHS^4^) [27] databases to evaluate the ECHS1 gene in the context of its association to diseases, functional associations with biological entities, human phenotypes, and pathways.

#### Association of ECHS1 with diseases

We used the Harmonizome database [26] which employs the standardized values related to the empirical *p*-value as abs(standardized value) = −log_10_(p-value) to determine associations between disorders and the ECHS1 gene. To further assess whether there is a link between the ECHS1 gene and the cardiovascular disease phenotypes, a dataset with 2684 genes was used. This dataset was produced through text-mining GWAS publications from the HuGE Navigator Gene-Phenotype Associations dataset. Data showed that the relative strength of association between the ECHS1 gene and cardiovascular diseases was -log_10_(p-value) = 2.03394. The complete table can be found at <http://amp.pharm.mssm.edu/Harmonizome/gene_set/Cardiovascular+Diseases/CTD+Gene-Disease+Associations>.

A dataset containing 2756 genes/proteins associated with cardiomyopathies from the curated CTD (Comparative Toxicogenomics Database) Gene-Disease Associations dataset was used to evaluate the link between the ECHS1 gene and cardiomyopathy. Data showed that the relative strength of association between the ECHS1 gene and cardiomyopathy was -log_10_(p-value) = 1.62423. The complete table can be found at <http://amp.pharm.mssm.edu/Harmonizome/gene_set/Cardiomyopathies/CTD+Gene-Disease+Associations>.

To evaluate whether there is a relationship between the ECHS1 gene and heart failure 2994 genes/proteins associated with Heart Failure from the curated CTD Gene-Disease Associations dataset was used. Results showed that the relative strength of association had a -log_10_(p-value) = 1.60734. The complete table can be found at <http://amp.pharm.mssm.edu/Harmonizome/gene_set/Heart+Failure/CTD+Gene-Disease+Associations>.

We also wanted to see if the ECHS1 gene was associated with other drug-induced conditions. Data from a dataset of 13,896 genes/proteins from the curated CTD Gene-Disease Associations showed that there is a relationship between the ECHS1 and drug-induced liver Injury (−log_10_(p-value) = 2.45226). The complete table can be found at < http://amp.pharm.mssm.edu/Harmonizome/gene_set/Drug-Induced+Liver+Injury/CTD+Gene-Disease+Associations>.

Results from the curated CTD Gene-Disease Associations dataset containing 7152 genes/proteins showed an association between the ECHS1 gene with Drug-Related Side Effects and Adverse Reactions (−log_10_(p-value) = 2.13125). Complete results can be obtained at < http://amp.pharm.mssm.edu/Harmonizome/gene_set/Drug-Related+Side+Effects+and+Adverse+Reactions/CTD+Gene-Disease+Associations>.

#### Relationships between ECHS1 with drugs

We used the comparative toxicogenomics database at <http://ctdbase.org/detail.go?type=gene&acc=1892&view=ixn>, http://ctdbase.org/detail.go?acc=1892&view=chem&page=2&type=gene>, and <http://ctdbase.org/detail.go?acc=1892&view=disease&sort=networkScore&type=gene&dir=asc> to evaluate if and how chemical interactions with the ECHS1 gene affect its expression and to assess chemical interference in the contexts of diseases associated with the ECHS1 gene. Data showed additional evidence that the ECHS1 gene not only interacts with different chemicals (Additional file 1: Tables S18 and S19) but also interferes with many (Additional file 1: Table S20).

#### ECHS1 functional predictions

We used the All RNA-seq and ChIP-seq Sample and Signature Search (ARCHS^4^) database to evaluate ECHS1 functional associations with biological entities. [27] Data showed that there is a relationship between the ECHS1 gene with known members of the branched-chain amino acid catabolic process gene set (z-score = 4.74044801), fatty acid beta-oxidation (GO:0006635) (z-score = 4.69401918), mitochondrial ATP synthesis coupled proton transport (GO:0042776) (z-score = 4.58179795), short-chain fatty acid metabolic process (GO:0046459) (z-score = 4.48892724), fatty acid oxidation (GO:0019395) (z-score = 4.39638234), lipid oxidation (GO:0034440) (z-score = 4.32665875), valine metabolic process (GO:0006573) (z-score = 4.32524268), branched-chain amino acid metabolic process (GO:0009081) (z-score = 4.27657735), organic acid catabolic process (GO:0016054) (z-score = 3.93805682), energy coupled proton transport, down electrochemical gradient (GO:0015985) (z-score = 3.90139956), ATP synthesis coupled proton transport (GO:0015986) (z-score = 3.90139956), respiratory electron transport chain (GO:0022904) (z-score = 3.63663297), very long-chain fatty acid metabolic process (GO:0000038) (z-score = 3.33068462), oxidative phosphorylation (GO:0006119) (z-score = 3.32720532), and drug metabolic process (GO:0017144) (z-score = 3.25059831), and drug catabolic process (GO:0042737) (z-score = 3.57297358). The complete table can be found at <https://amp.pharm.mssm.edu/archs4/gene/ECHS1>.

**Fig. 19:**
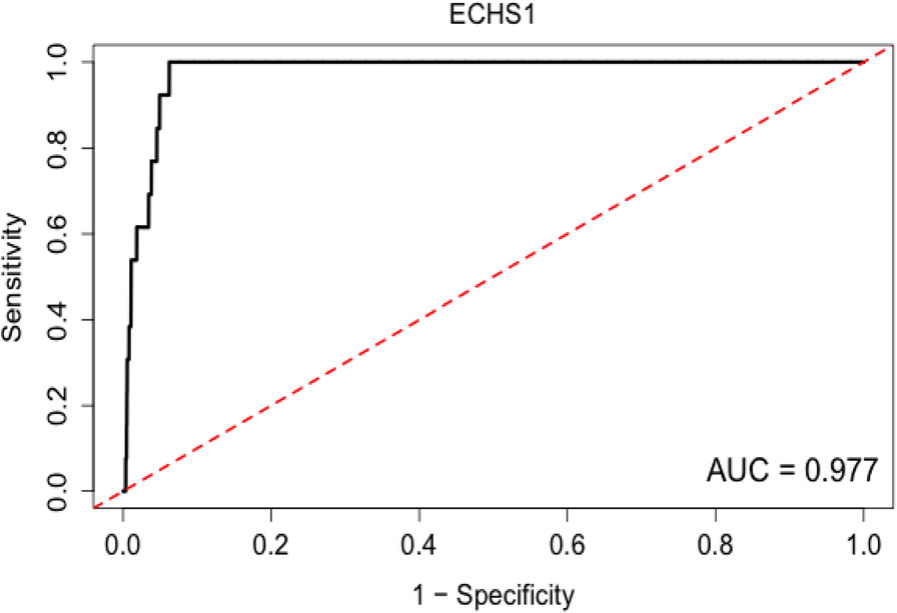
ECHS1 and functional prediction ROC curve

Results also show that the ECHS1 gene is extensively annotated with an AUC = 0.977, indicating how known annotations could be retrieved by the ARCHS^4^ algorithm (Fig. 19). In these analyses, gene set membership is calculated using membership by association. If a gene shares large associations with identified members of a gene set, it will receive a high z-score during the membership calculation. If a gene already has known functions/gene set memberships, they are printed in green and if a gene is widely annotated a ROC curve is generated.

#### ECHS1 predicted human phenotypes

Results also show that there is an association between the ECHS1 gene and Ketosis (HP:0001946) (z-score = 4.61091909), Ketoacidosis (HP:0001993) (z-score = 3.97801970), abnormality of long-chain fatty-acid metabolism (HP:0010964) (z-score = 3.92120687), metabolic acidosis (HP:0001942) (z-score = 3.21681082), and abnormality of fatty-acid metabolism (HP:0004359) (z-score = 4.67735644). Data also showed a relationship between the ECHS1 gene and abnormal activity of the mitochondrial respiratory chain (HP:0011922) (z-score = 2.83543187) and decreased activity of mitochondrial respiratory chain (HP:0008972) (z-score = 2.83543187). Evaluations of the ECHS1 gene also showed that the gene is associated with increased muscle lipid content (HP:0009058) (z-score = 2.59355360), and abnormal mitochondria in muscle tissue (HP:0008316) (z-score = 2.43905932). Further evaluations showed that the ECHS1 gene is related with right ventricular cardiomyopathy (HP:0011663) (z-score = 2.04008448), cardiovascular calcification (HP:0011915) (z-score = 2.05269516), sudden death (HP:0001699) (z-score = 2.05545235), progressive muscle weakness (HP:0003323) (z-score = 1.83832924), ragged-red muscle fibers (HP:0003200) (z-score = 2.36338320), and increased muscle lipid content (HP:0009058) (z-score = 2.59355360). The complete table can be found at <https://amp.pharm.mssm.edu/archs4/gene/ECHS1>.

#### ECHS1 predicted pathways (KEGG)

**Fig. 20:**
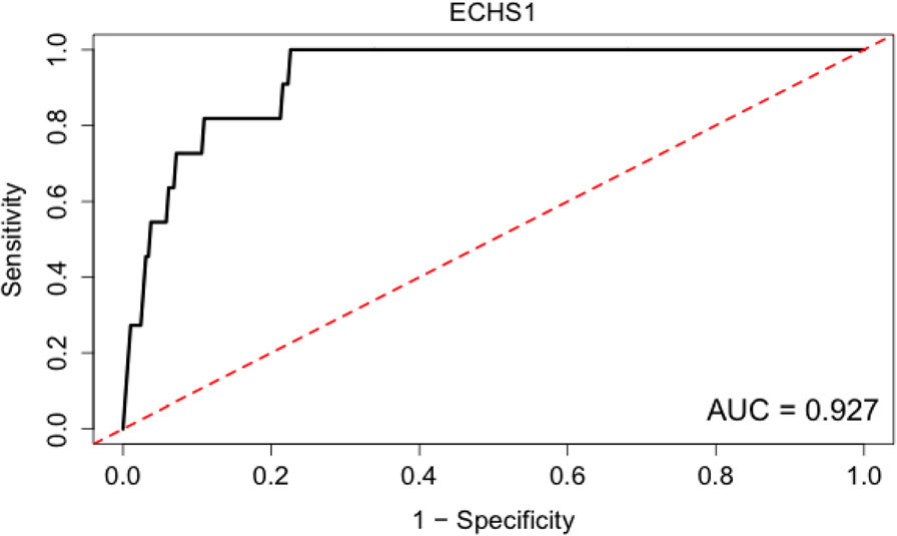
ECHS1 and predicted KEGG pathway ROC curve

Results also show that there is an association between the ECHS1 gene with valine, leucine and isoleucine degradation_Homo sapiens_hsa00280 (z-score = 3.1786154), fatty acid degradation_Homo sapiens_hsa00071 (z-score = 3.04847658), and fatty acid metabolism_Homo sapiens_hsa01212 (z-score = 2.51812360). Interestingly, results also show that there is a relationship between the ECHS1 gene with drug metabolism - cytochrome P450_Homo sapiens_hsa00982 (z-score = 2.07985196). The complete table can be found at <https://amp.pharm.mssm.edu/archs4/gene/ECHS1>. Additionally, results again show that the ECHS1 gene is extensively annotated with an AUC = 0.927, indicating how known annotations could be retrieved by the ARCHS^4^ algorithm (Fig. 20).

## Discussion

We report an innovative filtering method that integrates RNA-Seq variant detection and transcriptome analysis, with pathway analysis that is useful in identifying putative pathogenic and modifying pathways, mutations, and effect modifier genes that may be involved in triggering drDCM in patients who are not related. The most significant findings are that there is a strong association between drDCM with fatty acid and branched-chain amino acid metabolism and that this relationship may arise from an interplay between the putative mutation *ECHS1*:11 V/A(rs10466126), modifiers *ECHS1*:75 T/I(rs1049951), *ECHS1*:c.41insT, and *DBT*:384G/S(rs12021720), upregulation of ECHS1, the MIR3944 mirtron, and DBT, and the involvement of ECHS1 in the drug metabolism: cytochrome P450 pathway.

Huang et al. 2011 wondered whether “branched-chain amino acid metabolism in heart disease” was an “epiphenomenon or a real culprit.” [28] Authors make statements that intermediates that take part in the breakdown of branched-chain amino acids (BCAAs) may initiate cardiac dysfunction. Others have reported genetic disorders that arise when there are defects in the BCAA catabolic pathways. These conditions include propionic acidemia, methylmalonic acidemia, and Maple syrup urine disease. The clinical symptom associations with these disorders are mostly mental retardation and seizure [28–30] although others have already connected methylmalonic and propionic academia to hypertrophic and dilated cardiomyopathy [23, 29, 31–34]. In the disease state, flaws in the use of glucose and the inhibition of fatty acid oxidation in myocytes lead to the insufficient delivery of energy to the heart muscle [35, 36]. Hence, it is evident, that there is a high probability that pathways associated with fatty acid β-oxidation, and the branched chain amino acids and their intermediate substrates contribute to the pathogenesis of heart disease.

### Association between drDCM and ECHS1

The *ECHS1* gene (cytogenetic position: 10q26.3; OMIM*602292; GenBank accession number: NM_004092.3) produces the Short-chain enoyl-CoA hydratase (SCEH, synonym: crotonase, EC 4.2.1.17). It is an enzyme found in the mitochondrial matrix that catalyzes the second step in the mitochondrial fatty acid beta-oxidation. [37] It also takes part in the valine and isoleucine catabolic pathways. For instance, in valine catabolism, it functions upstream HIBCH (3-Hydroxyisobutyryl-CoA Hydrolase) and converts acryloyl-CoA and methacrylyl-CoA to (S)-3-hydroxyisobutyryl-CoA to 3-hydroxypropionyl-CoA [38]. ECHS1 catalyzes fatty acids that are saturated and have single bonds. It catalyzes the addition of H_2_O (water) to the trans bond of the Δ2-enoyl-CoA created during β-oxidation [39].

### *ECHS1*:rs10466126 and *ECHS1*:rs1049951

In this study, there is an association between ECHS1 and drDCM and carries two common missense variants, *ECHS1*:rs10466126, a putative mutation and the *ECHS1*:rs1049951 and the *ECHS1*:c.41insT putative effect modifiers. Some might argue that our methods are linking common variants to drDCM since DCM is not common in the general population. However, in our data subjects carry at least two variants that interact. Further linkage disequilibrium evaluations of *ECHS1*:rs10466126 and *ECHS1*:rs1049951 reveal that these two genes do not just interact, they are also co-inherited. Thus, the frequency of having at least two variations in the *ECHS1* gene in the general population would be much lower than having only one.

Additionally, data offered by Chen et al. 2015 demonstrated an association between the rs7597774 mutation in the ADD2 (Beta-adducin) gene, a common variant (A = 0.3928 (1000 Genomes)) and DCM [40]. Accordingly, our data augment such results and suggest that not only are rare variants associated with DCM, SNPs that are common also play a significant role. Moreover, transcriptome analyses show that the EGR1 transcription factor is down-regulated, suggesting that the rate at which it is binding to the 5’-GCG(T/G)GGGCG-3′(EGR-site) DNA sequence to regulate *ECHS1* is also reduced. According to JASPAR prediction analyses, when these transcription regulators bind the GCG(T/G)GGGCG-3′(EGR-site) motif, they bind more frequently to the alternative allele (G) (Fig. 8). Interestingly, our data not only show variation at the DNA and mRNA level, but there are also perturbations at the function level associated with the EGR1 transcription regulator. Cardiovascular system development and function and development of endothelial tissue is decreased. Together, these findings indicate that *ECHS1*:rs10466126 (G/G), even though it may be tolerated as an exonic variant, as an upstream variant, it might be interfering with the transcriptional regulation of *ECHS1* by EGR1.

As mentioned above, our findings show that the alternative allele is the (G) for the *ECHS1*:rs10466126 missense, upstream variant 2 KB, putative mutation. However, this allele is currently the more frequent in the general population, and the ancestral allele (A), which is conserved among species is less common (Fig. 7). However, when one stratifies by population it is evident that this allele is less frequent in certain regions like Africa (Additional file 1: Table S12).

When one considers disease selection and allele frequency from a genetic perspective, one can characterize evolution as a change in allele frequencies over a period because of genetic drift, natural selection, migration, or mutation [41]. Recent shifts in the direction of selection could contribute to segregation of disease alleles at moderate or high frequencies. For example, the popular thrifty-genotype hypothesis [42] states that selection has formerly worked to take advantage of metabolic effectiveness, particularly in people that frequently run into a shortage of food. Dietary changes could reverse the direction of selection as well, which could cause common alleles that are currently associated with metabolic diseases and diabetes to be selected against. Genetic hitchhiking [43]. during a selective sweep, for example, could result in disease genes to be associated with a positive selection [44].

It appears some evolutionary force has occurred that has changed the allele frequency of the *ECHS1*:rs10466126 variants in some populations. This observation parallels current studies showing over 40% of U.S. citizens ages 60 and older have metabolic syndrome. [45, 46] This syndrome is characterized by a collection of metabolic aberrations that increase the chance of developing the atherosclerotic cardiovascular disease and Type 2 diabetes [47]. The marked rise in the prevalence of the metabolic syndrome in the past two decades matches the global epidemic of diabetes and obesity, as well [48]. In their report, Weiss et al. indicated that the prevalence of the metabolic syndrome in the U.S.A. rose with how severe obesity was and reached 50% in adolescents who were severely obese [49].

### The ECHS1 gene

In this study, the ECHS1 gene is the main drDCM driver, and we evaluate its involvement in diseases, biological functions, and pathways in multiple datasets from the ARCHS^4^ and Harmonizome databases. Among the diseases, the ECHS1 gene contributes are cardiovascular diseases, cardiomyopathies, and heart failure. Interestingly, data also show that there is an association between the ECHS1 with drug-related side effects and adverse reactions (<http://amp.pharm.mssm.edu/Harmonizome/gene_set/Cardiomyopathies/CTD+Gene-Disease+Associations>, <http://amp.pharm.mssm.edu/Harmonizome/gene_set/Heart+Failure/CTD+Gene-Disease+Associations>, < http://amp.pharm.mssm.edu/Harmonizome/gene_set/Drug-Induced+Liver+Injury/CTD+Gene-Disease+Associations>, < http://amp.pharm.mssm.edu/Harmonizome/gene_set/Drug-Related+Side+Effects+and+Adverse+Reactions/CTD+Gene-Disease+Associations>). These findings are remarkable because there is a relationship between drDCM with one’s reaction to drugs and heart disease.

Further evaluations of ECHS1, revealed that this gene has functional relations with branched-chain amino acid catabolic processes, fatty acid beta-oxidation, valine metabolic process, branched-chain amino acid metabolic process, very long-chain fatty acid metabolic process, oxidative phosphorylation, and mitochondrial ATP synthesis coupled proton transport. Remarkably, results also show that there is an association between the ECHS1 gene with drug metabolic processes. These findings are noteworthy because, in our drDCM dataset, we found similar processes associated with the ECHS1 gene. However, in the ARCHS^4^ and Harmonizome databases, results show that there is also an association between the ECHS1 gene and processes related to drug metabolism.

When we evaluated the ECHS1 gene in the contexts of human phenotypes, we found that it is associated with ketoacidosis, abnormality of long-chain fatty-acid metabolism, abnormal activity of mitochondrial respiratory chain, increased muscle lipid content, abnormal mitochondria in muscle tissue, progressive muscle weakness, and cardiovascular calcification (<https://amp.pharm.mssm.edu/archs4/gene/ECHS1>). These findings again are similar to results from the drDCM dataset.

Further assessment of the ECHS1 gene in the framework of pathways (KEGG) revealed that this gene participates in valine, leucine, isoleucine, and fatty acid degradation, the same results obtained from the drDCM dataset. Consistent with the other results from the more extensive datasets, findings showed that the ECHS1 gene is also related to drug metabolism: cytochrome P450 (<https://amp.pharm.mssm.edu/archs4/gene/ECHS1>).

These additional assessments of the ECHS1 gene are intriguing and strengthen the findings from our drDCM dataset. They provide additional information and insight about why patients with mutations in this gene can be refractory to drugs. For example, our data show that the ECHS1 gene is related to the drug metabolism: cytochromes P450 pathway. Others have reported that cytochromes P450 comprise the key family of chemical modifications of the majority of drugs and other lipophilic xenobiotics in the body. [50–52]

### Association between ECHS1, BCAAs and LVLFAs

Using our integrative method, we show that the valine, isoleucine degradation 1 pathways, and the fatty acid β-oxidation pathways all carry ECHS1, which are all associated with the drDCM data set. Data suggest that ECHS1 is a hub that mediates pathogenic crosstalk amongst the three pathways. Isoleucine and valine are branched chain amino acids degraded and oxidized as fuel in extra-hepatic tissue that includes brain, kidney, adipose, and muscle. Other types of amino acids are broken down in the liver. Extrahepatic tissues have amino-transferase, an enzyme not found in liver. It catalyzes valine, isoleucine, and leucine to make the product α-keto acid. [39] Valine and isoleucine go through a sequence of reactions to generate propionyl-CoA for valine, and propionyl-CoA and acetyl-CoA for isoleucine. Interestingly, parts of the valine and isoleucine pathways closely parallel steps in the fatty acid β-oxidation pathway. [39]

Fatty acids and related lipids play an essential role in cardiomyocyte function and structure. In post-natal and adult mammalian heart, fatty acid β-oxidation is the favored pathway used to generate the energy needed for efficient pumping of the heart. Acquired or inherited defects in the mitochondrial fatty acid metabolism may result in arrhythmias and cardiomyopathy that predisposes patients to heart failure. [39] Their effect on the stability and fluidity of the structure of membranes affects their function as transporters of ions and substrates. It also affects the electrophysiology that is fundamental to heart function and heart excitation. Additionally, there are implications that fatty acids and related lipids regulate cell signaling, are effectors in apoptosis and responses to ischemic and oxidative damage and are second messengers in transduction. [39] In the process of fatty acid oxidation, β-oxidation takes place during the subsequent removal of carbons at the β-carbon site of the fatty acyl-CoA molecule producing NADH, FADH2 (flavin adenine dinucleotide), and acetyl-CoA downstream. This process generates more energy per carbon atom and uses more oxygen compared with the oxidation of carbohydrates. Thus, oxygen plays an essential role in ATP (adenosine triphosphate) utilization by the myocardium. [53]

As mentioned above, key enzymes in the degradation processes of the two amino acid pathways are up-regulated, suggesting that production of NADH, H+, acetyl-CoA, propionyl-CoA, 2-oxoglutarate, and (S)-3-amino-2-methyl-propanoate is increased and would accumulate in the mitochondria of the myocardium. The buildup of acetyl-CoA and propionyl-CoA may lead to a buildup of acetoacetate (a keto-acid) and propionic acid in the myocardium, respectively. In turn, accumulation of acetoacetate results in ketoacidosis, and accumulation of propionic acid may lead to propionic acidemia. [54–56] Accumulation of 2-oxoglutarate and (S)-3-amino-2-methyl-propanoate may very well be toxic to the myocardium (Additional file 6: Fig. S5).

We also report that ACSL5 (acyl-CoA synthetase long chain family member 5) and SLC27A3 (Solute carrier family 27 member 3) are down-regulated suggesting that there is no activation of long and very long chain fatty acids. Data indicates that there is a possible accumulation of long and very long chain fatty acids in the cytoplasm that could lead to toxicities in the myocardium leading to muscle damage.

### DBT and drDCM

In the valine, leucine, and isoleucine degradation 1 pathway *DBT* carries the rs12021720 variant, which interacts with the rs10466126 variant in the ECHS1 gene (Table 2). *DBT* encodes a protein that forms the critical homo-24-meric dihydrolipoyl transacylase (E2) subunit of the branched-chain alpha-keto acid dehydrogenase complex (BCKD), an enzyme complex that is on the inside of the mitochondria. This compound is known to catalyze the oxidative decarboxylation of branched-chain α-keto acids (BCKAs). It is not only the rate-limiting step, but is also an irreversible step of valine, isoleucine, and leucine catabolism [54]. The other subunits of the BCKD complex include the associated decarboxylase (E1) and the dehydrogenase (E3) regulatory subunits. Interestingly, the phosphorylation status of the E1α regulatory subunit of BCKD determines whether BCKD is active or not. When there is a depletion of BCAAs, a BCKD kinase hyper-phosphorylates BCKD resulting in inhibition of BCKD activity and conservation of free BCAA. However, when the levels of BCAA are high, a BCKD phosphatase dephosphorylates E1α resulting in the activation of BCKD and a reduction in total BCAA [54].

Mutations in DBT are known to cause Maple syrup urine disease, type 2. Mutations interrupt the normal function of the E2 subunit, which in turn prevents the BCKD enzyme complex from breaking down the amino acids efficiently. These results lead to the accumulation of BCAA and their byproducts, which end up poisoning the cells and tissues, and impairing vital organs, including the heart [54–56].

We report that there is an up-regulation of DBT and the other subunits of the BCKD enzyme complex and that there is an association between drDCM and DBT (Additional file 6: Fig. S5) at the mRNA levels. These results suggest that perturbations to DBT contribute to increased rates at which the branched chain amino acids are being broken down and the subsequent accumulation of organic acid products in the mitochondria.

### MCCC1 and drDCM

The *MCCC1* gene produces the alpha subunit of the 3-methylcrotonoyl-CoA carboxylase complex (3-MCC) found in the mitochondria. It interacts with the beta subunits made from the *MCCC2* gene and the B-vitamin biotin to form a functioning enzyme. [56] The 3-MCC compound converts one 3-methylcrotonyl-CoA to 3-methylglutaconyl-CoA in the fourth step of leucine degradation (Additional file 6: Figure S5). There is an activation of the 3-MCC complex caused by upregulation of MCCC1 and MCCC2 in cases vs. controls. Interestingly, in the cytoplasm, HMGCLL1 (3-hydroxymethyl-3-methylglutaryl-CoA lyase like 1) is down-regulated (Additional file 1: Table S14). This enzyme converts (S)-3-hydroxy-3-methylglutaryl-CoA into acetyl- CoA and acetoacetate, in the cytoplasm. Down-regulation of this enzyme in our data set suggests that the rate at which (S)-3-hydroxy-3-methylglutaryl-CoA is converted into acetyl-CoA, and acetoacetate is reduced and that there could be an accumulation of (S)-3-hydroxy-3-methylglutaryl-CoA in the cytoplasm. Interestingly, there is also up regulation of a related molecule, HMGCL (Hydroxymethylglutaryl-CoA Lyase) in the mitochondria that catalyze a similar reaction. Thus, there would be a possible accumulation of (S)-3-hydroxy-3-methylglutaryl-CoA in the cytoplasm and buildup of acetyl- CoA and acetoacetate in the mitochondria (Additional file 6:Figure S5).

#### ECHS1 and mitochondrial dysfunction and actin cytoskeleton

Perturbations in the fatty acid β-oxidation, isoleucine, and valine pathways are associated with *ECHS1*:rs10466126(G/G) at the DNA, mRNA and pathway levels. Association of ECHS1 with drDCM is modified by the presence of DBT leading to mitochondrial, oxidative phosphorylation and TCA cycle dysfunction through organic acid toxicity in the mitochondria and lipotoxicity in the cytoplasm of the myocardium (Additional file 1: Figure S5). Furthermore, signals from the 26 s proteasome and NFκB complexes act on the mitochondrial, and through interactions with EPS8 (Epidermal growth factor receptor kinase substrate 8), a signaling adapter and PALLD (Palladin), a cytoskeletal protein, deleterious signals reach the actin cytoskeleton, which affects the optimum function of the heart.

### Proposed molecular pathogenesis of drDCM

We proposed that patients with drDCM have disruptions to fatty acid and BCAA catabolism arising from genomic errors in ECHS1, which is modified by DBT. Perturbations in these pathways lead to the buildup of protons, NADH, acetyl-CoA, propionyl-CoA and leucine, valine, fatty acid, and isoleucine intermediates in the mitochondria, giving rise to ketoacidosis and propionic acidemia, and additional organic acid toxicity. The buildup of long and very long chain fatty acids and leucine intermediates in the cytoplasm may lead to lipotoxicity and organic acid toxicity. These physiological conditions lead to mitochondrial, oxidative phosphorylation and TCA cycle II dysfunction.

**Fig. 21:**
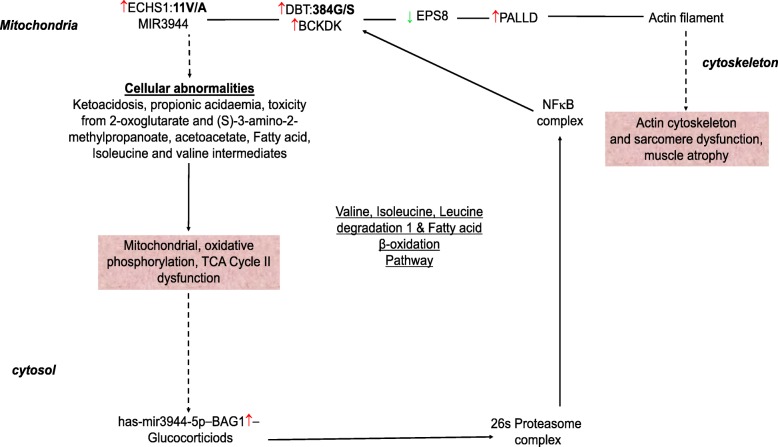
Model of mitochondrial dysfunction in patients with drDCM. Legend: Doted lines indicate several processes take place and solid lines direct interactions or outcomes. Red arrows pointing up indicate up-regulated genes. Green arrows pointing down indicate down-regulated genes. In bold are variants found in the respective genes

Interestingly, in another study we found that BAG1 indirectly interacts with ECHS1 through the 26 s Proteasome and the NFκB complex. Dysfunction in the mitochondria recruits BAG1:Glucocorticoids, which translocate to the mitochondria, where the Glucocorticoids interact with and regulate the E2 subunit, where other BCKAD subunits assemble at the transcriptional level. On the other hand, crosstalk between a dysfunctional mitochondria and cytoskeleton through PALLD contributes to insufficient supply of ATP to the actin cytoskeleton leading to muscle atrophy and sarcomere dysfunction (Fig. 21).

### Study strengths and limitations

We began our investigations by analyzing free open source secondary RNA-Seq and exome sequence data sets from the SRA using Apps from BaseSpace. We generated variant lists and gene lists with differentially expressed genes. We then integrated variants, differentially expressed genes and pathways, followed by variant replication and association analyses. The aim was to provide a novel approach for data integration that considers the interplay between DNA, mRNA and pathway analyses that can reveal mutations, effect modifiers, and pathogenic pathways. Another aim was to provide a method that can be used to model complex traits while uncovering genomic biomarkers and targets for early diagnosis, prognosis, and personalized therapeutic intervention.

#### Strengths

Our study casts light on the possibility that one gene in concert with effect modifier genes can mediate pathogenic crosstalk among multiple pathways. It also demonstrates that integrating RNA-Seq gene profiling and variant detection is a versatile approach to tease out genes that are causal from those that are effect modifiers. Discovery of an already known pathogenic variant in DBT, a differentially expressed gene provides added confidence that my filtering approach is efficient and that it holds real biological significance. Our study is robust. We used exome sequence data sets to determine whether the RNA-Seq variants of interest did not arise from RNA sequencing errors and that they are also found in other patients with DCM. Replicating the variant analyses in an independent and much larger sample size also strengthens this study, by establishing consistency. However, some issues stay unexplored by our research.

#### Limitations

Our results are limited by a small sample size and may not have detected small differences between cases and controls. While the case replication sample size is large (*n* = 128), the control replication sample size is only *n* = 15. Such a small size may have affected the power to detect small effects between the two groups.

By using RNA-Seq data, differentially expressed genes (q < 0.05), variants found in differentially expressed genes that are statistically associated with the outcome (*p* < 0.05) and located in pathways and biological functions associated with the data sets (−log (p) ≥ 1.3), we have increased the biological power significantly. It is also important to note that DCM is rare in the population. To study the condition using RNA-Seq involves extracting tissue samples from the heart, an invasive procedure. Thus, finding cases and the proper controls would be a limitation in many similar studies.

Moreover, we were unable to control for potential confounders that might have contributed to some of the observed differently expressed transcripts among cases and controls. For example, drugs might have interfered with gene expression. Additionally, RNA-Seq variant detection is biased towards coded regions, which do not embody the location of all causative mutations. This restriction also exists in present exome sequence DCM-gene panels, suggesting that both current and surfacing genome-targeted approaches that do not investigate the whole genome will not attain 100% sensitivity to diagnose all genomic forms of DCM [15].

## Conclusion

We conclude that patients with drDCM carry the *ECHS1*:11 V/A (rs10466126) putative mutation that interacts with the *ECHS1*:75 T/I (rs1049951) and DBT:384G/S (rs12021720) putative modifiers, which are associated with drDCM. *ECHS1*:11 V/A (rs10466126) and *ECHS1*:75 T/I (rs1049951) are also co-inherited. The ECHS1 gene hosts a mirtron, MIR3944 that is expressed in drDCM patients and not in controls. The ECHS1 gene is not only associated with heart disease, it is also associated with drug metabolism. We also present the first use of an integrative “omics” data approach that considers the interplay between the DNA, mRNA, and drDCM related pathways. Our method can discriminate mutations from modifiers, and pathogenic pathways from pathways that modify the outcome. Our technique represents a potential diagnostic, prognostic, biomarker, and treatment discovery methodology in a genomically heterogeneous disorder like drDCM.

### Translational perspective

Our integrative approach will be useful in modeling complex traits while revealing genomic biomarkers and targets for therapeutic intervention. The method will also be valuable for the discovery of diagnostic and prognostic tools that consider the interplay between DNA, mRNA, and related pathways. The results revealed raise the potential for MIR3944 to be both a diagnostic and prognostic biomarker for drDCM. Data show that MIR3944 is expressed in patients with drDCM but not in the controls suggesting that the expression of MIR3944 in the heart is unusual and indicative of drDCM. Because MIR3944 is not detected in healthy tissue, it would serve as a more precise and sensitive biomarker then ECHS1 which is also expressed in the healthy tissue, for example. While overexpression of ECHS1 is suggestive of drDCM, a detectable limit at which to determine what levels are pathogenic would first have to be established for each patient. However, for MIR3944, the mere detection in a patient would indicate pathology. ECHS1 could be targeted for the design of a novel drug, and elimination of BCAAs and LVLFAs from one’s diet could repair three drDCM pathogenic pathways simultaneously. One could also use levels of Glucocorticoids, acetoacetate, propionic acid, and fatty acids as biomarkers for drDCM. ECHS1 could be used, as a biomarker to implement risk stratification that improves the management of drDCM. ECHS1 protein may be a potential therapeutic target for drDCM patients who are not related.

## Additional files

Additional file 1: **Table S1.** Exome sequence data sets from the HapMap. Legend: Exome sequencing of (CEU) Utah residents with ancestry from Northern and Western Europe and of (CHB) Han Chinese in Beijing, China - CEPH – HapMap. **Table S2.** Coverage: Reads mapped per drDCM subject. Legend: Percentage and number of reads mapped before and after filtering for each subject in the drDCM case control study. **Table S3.** Alignment Summary (Italian). Legend: Mean coverage: the total number of targeted bases divided by the targeted region size. Target coverage at 1X: Percentage targets with coverage greater. **Table S4.** Alignment summary (Chinese). Legend: Mean coverage: the total number of targeted bases divided by the targeted region size. Target coverage at 1X: Percentage targets with coverage greater. **Table S5.** Alignment summary: HapMap project data set. Legend: Mean coverage: the total number of targeted bases divided by the targeted region size. Target coverage at 1X: Percentage targets with coverage greater than 1X. Target coverage at 10X: Percentage targets with coverage greater than 10X. Target coverage at 20X: Percentage targets with coverage greater than 20X. Target coverage at 50X: Percentage targets with coverage greater than 50X. **Table S6.** drDCM 131 variants in differentially expressed genes. **Table S7.** drDCM Pathways. **Table S8.** Variants found in a pathway. **Table S9.** RNA-Seq drDCM genotypes: ECHS1, DBT, and MCCC1. Legend: Ref: reference allele, Alt: alternative allele, DCM: dilated cardiomyopathy, CTR: control. ECHS1: enoyl-CoA hydratase, short chain, 1, mitochondrial, DBT: Dihydrolipoamide branched chain transacylase E2, and MCCC1: methyl crotonoyl-CoA carboxylase 1. **Table S10.** Variants in DCM pedigrees from Italy and China. Legend: Genotypes for DCM cases for the ECHS1, DBT, and MCCC1 genes. ECHS1: enoyl-CoA hydratase, short chain, 1, mitochondrial, DBT: Dihydrolipoamide branched chain transacylase E2, and MCCC1: methyl crotonoyl-CoA carboxylase 1. **Table S11.** Variant scanning in HapMap data set. Legend: Control genotypes for the ECHS1, DBT, and MCCC1 genes. ECHS1: enoyl-CoA hydratase, short chain, 1, mitochondrial, DBT: Dihydrolipoamide branched chain transacylase E2, and MCCC1: methyl crotonoyl-CoA carboxylase 1. **Table S12.** Population Genetics for the ECHS1:rs10466126 Putative Mutation. Legend: Population Code: “CHB: Han Chinese in Beijing, China, JPT:Japanese in Tokyo, Japan, CHS: Southern Han Chinese, CDX: Chinese Dai in Xishuangbanna, China, KHV: Kinh in Ho Chi Minh City, Vietnam, CEU: Utah Residents (CEPH) with Northern and Western European Ancestry, TSI: Toscani in Italia, FIN: Finnish in Finland, GBR: British in England and Scotland, IBS: Iberian Population in Spain, YRI: Yoruba in Ibadan, Nigeria, LWK: Luhya in Webuye, Kenya, GWD: Gambian in Western Divisions in the Gambia, MSL: Mende in Sierra Leone, ESN: Esan in Nigeria, ASW: Americans of African Ancestry in SW USA, ACB: African Caribbeans in Barbados, MXL: Mexican Ancestry from Los Angeles USA, PUR: Puerto Ricans from Puerto Rico, CLM: Colombians from Medellin, Colombia, PEL: Peruvians from Lima, Peru, GIH: Gujarati Indian from Houston, Texas, PJL: Punjabi from Lahore, Pakistan, BEB: Bengali from Bangladesh, STU: Sri Lankan Tamil from the UK, ITU: Indian Telugu from the UK.” (<http://www.internationalgenome.org/category/population/>). **Table S13.** Novel ECHS1 c.41insT. **Table S14.** drDCM differentially expressed genes. **Table S15.** drDCM Diseases and Functions. **Table S16.** ECHS1:rs10466126 and ECHS1:rs1049951 pairwise linkage disequilibrium in 24 populations from the 1000 genomes project. **Table S17.** Data mining: IPA knowledge database, PALLD. **Table S18.** Chemicals that interact with the ECHS1 gene. **Table S19.** The effect of chemical interactions on the expression of the ECHS1 gene. **Table S20.** Chemicals associated with diseases that interfere with the ECHS1 gene. (XLSX 632 kb)

Additional file 2: **Figure S1.** Transcription factors that bind to the ECHS1 gene. (PPTX 202 kb)

Additional file 3: **Figure S2.** Expression profiles for ECHS1 and has-mir-3944 in normal heart. Legend: Panel 1: ECHS1 expression profiles in 6 different tissues. Panel 2: has-mir-3944 expression profiles in 6 different tissues. Panel 3: Expression correlations between has-mir-3944-5p and the ECHS1 in the 6 different tissues. (PPTX 248 kb)

Additional file 4: **Figure S3.** Predicted conservation: Putative target region and has-mir-3944. Legend: **A** Conservation for RHOD. **B** Conservation for ITGAV. **C** Conservation for BAG1. (PPTX 545 kb)

Additional file 5: **Figure S4.** Pathways associated with the ECHS1 gene. (PPTX 849 kb)

Additional file 6: **Figure S5.** Proposed schematic: Dysfunction in the mitochondria. Legend: Arrows (red) pointing up represent up-regulated genes and arrows (green) pointing down, down-regulated genes. Printed in blue indicates putative modifiers and in red mutations. In bold print indicates genes carrying a variant. Orange arrows indicate the flow of catalysis in the fatty acid beta-oxidation pathway. Green arrows show the flow of catalysis in the Leucine degradation 1 pathway. Purple arrows show the flow of catalysis in the Valine degradation 1 pathway. Blue arrows indicate the flow of catalysis in the Isoleucine degradation 1 pathway. In boxes at the end of each pathway are proposed products of each pathway. (PPTX 583 kb)

## Abbreviations

ACSL5: acyl-CoA synthetase long-chain family member 5
ADD2: Beta-adducin
App: application
ARVC: Arrhythmogenic right ventricular cardiomyopathy
ATP: Adenosine triphosphate
BAG3: Bcl2-associated athanogene 3
BCAAs: Branched-chain amino acids
BCAAs: Branched-chain amino acids
BCKAs: Branched-chain α-keto acids
BCKD: Branched-chain alpha-keto acid dehydrogenase complex
BCKD: Branched-chain alpha-keto acid dehydrogenase complex
BCKDHA: Branched chain keto acid dehydrogenase E1, alpha polypeptide
BCKDHB: Branched chain keto acid dehydrogenase E1, beta polypeptide
bp: Base pairs
cDNA: Complementary DNA
CoA: Coenzyme A
CTR: Control
dbSNP: Single Nucleotide Polymorphism database
DBT: Dihydrolipoamide branched chain transacylase E2
DCM: Dilated cardiomyopathy
DLD: Dihydrolipoamide dehydrogenase
DNA: Deoxyribonucleic acid
E1: Decarboxylase
E2: Homo-24-meric dihydrolipoyl transacylase
E3: Dehydrogenase
ECHS1: Enoyl-CoA hydratase, short chain 1
ECHS1: Enoyl-CoA hydratase, short chain, 1, mitochondrial
EDGC: Eone-Dianomics Genome Center
FADH2: Flavin adenine dinucleotide
FDR: False discovery rate
FPKM: Fragments per kilobase of transcript per million mapped reads
gVCF: Genomic VCF
H +: Proton
H2O: Water
HCM: Hypertrophic cardiomyopathy
HIBCH: 3-Hydroxyisobutyryl-CoA Hydrolase
IPA: Ingenuity Pathway Analysis
Log: Logarithm
MAF: Minor allele frequency
mRNA: Messenger RNA
NADH: Nicotinamide adenine dinucleotide
Nextgen: Next generation
*p*-value: Probability value
q-value: FDR adjusted *p* -value
Rhod: Rho-related GTP-binding protein
RNA: Ribonucleic acid sequence
RNA-Seq: Ribonucleic acid sequence
SCEH: Short-chain enoyl-CoA hydratase
SLC27A3: Solute carrier family 27 member 3
SNPs: Single Nucleotide Polymorphisms
SRA: Sequence Read Archive
UTR: Untranslated regions
VEP: Variant Effect Predictor
