# Supplementary figures and images for: “Omics” data integration and functional analyses link Enoyl-CoA hydratase, short chain 1 to drug refractory dilated cardiomyopathy
Source: BMC Med Genomics. 2018 Dec 12;11:110. doi: 10.1186/s12920-018-0439-6 (PMC6292014; doi:10.1186/s12920-018-0439-6)

## Slide 1
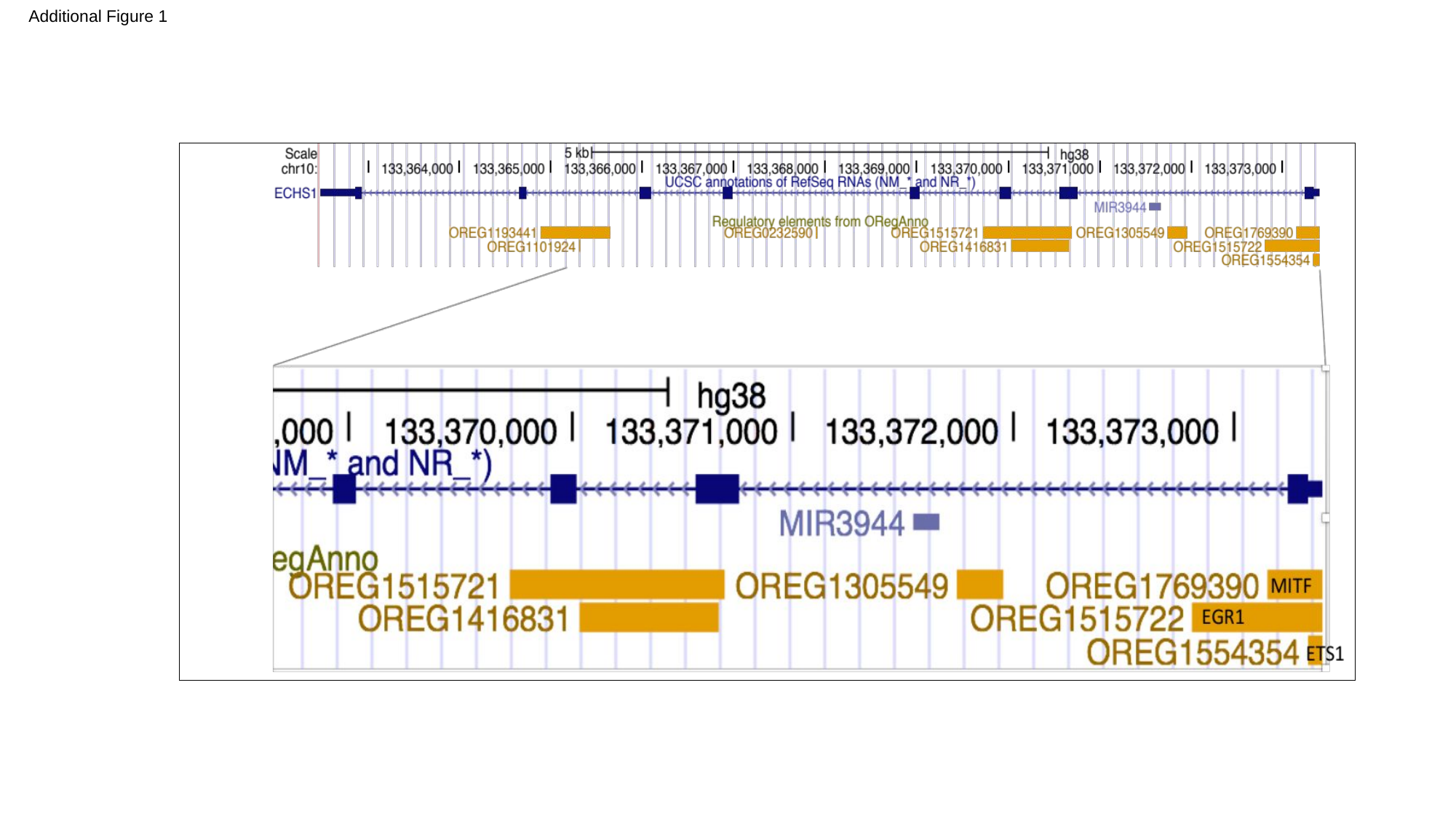

Additional Figure 1

Supplement: Supplementary file 2 — Figure S1. Transcription factors that bind to the ECHS1 gene. (PPTX 202 kb) [file 12920_2018_439_MOESM2_ESM.pptx]

## Slide 1
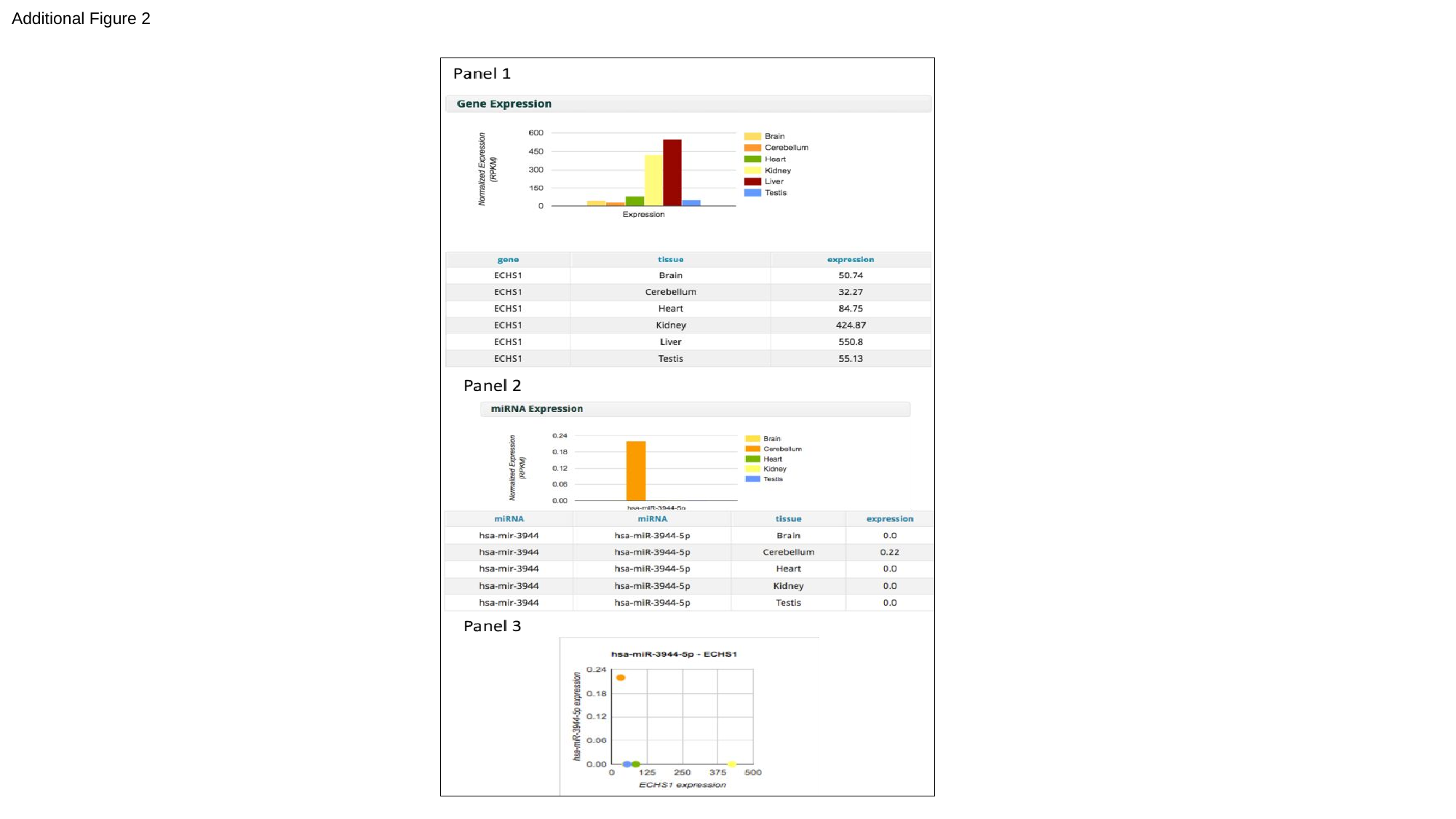

Additional Figure 2

Supplement: Supplementary file 3 — Figure S2. Expression profiles for ECHS1 and has-mir-3944 in normal heart. Legend: Panel 1: ECHS1 expression profiles in 6 different tissues. Panel 2: has-mir-3944 expression profiles in 6 different tissues. Panel 3: Expression correlations between has-mir-3944-5p and the ECHS1 in the 6 different tissues. (PPTX 248 kb) [file 12920_2018_439_MOESM3_ESM.pptx]

## Slide 1
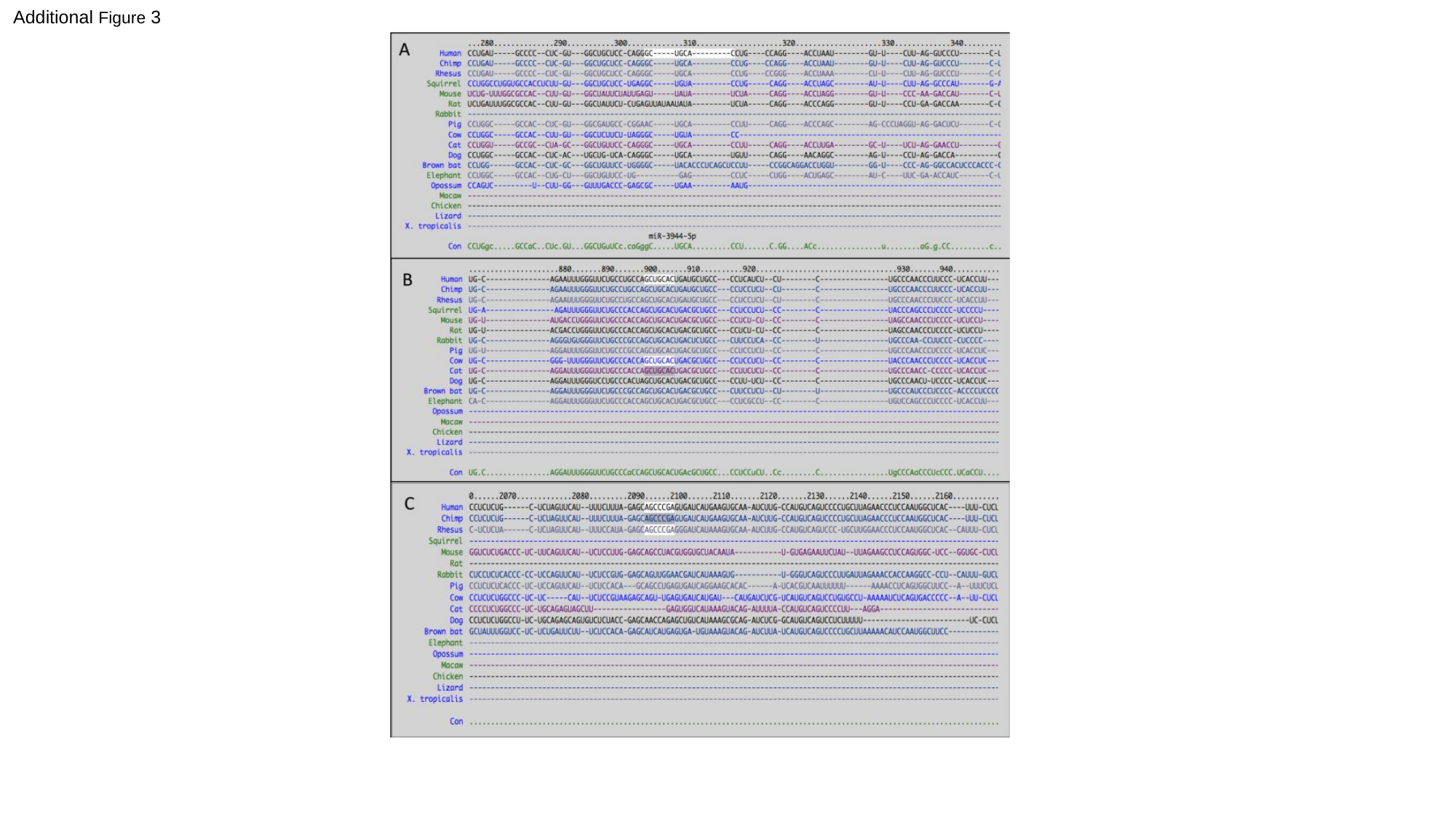

Additional Figure 3
Additional Figure 3

Supplement: Supplementary file 4 — Figure S3. Predicted conservation: Putative target region and has-mir-3944. Legend: A Conservation for RHOD. B Conservation for ITGAV. C Conservation for BAG1. (PPTX 545 kb) [file 12920_2018_439_MOESM4_ESM.pptx]

## Slide 1
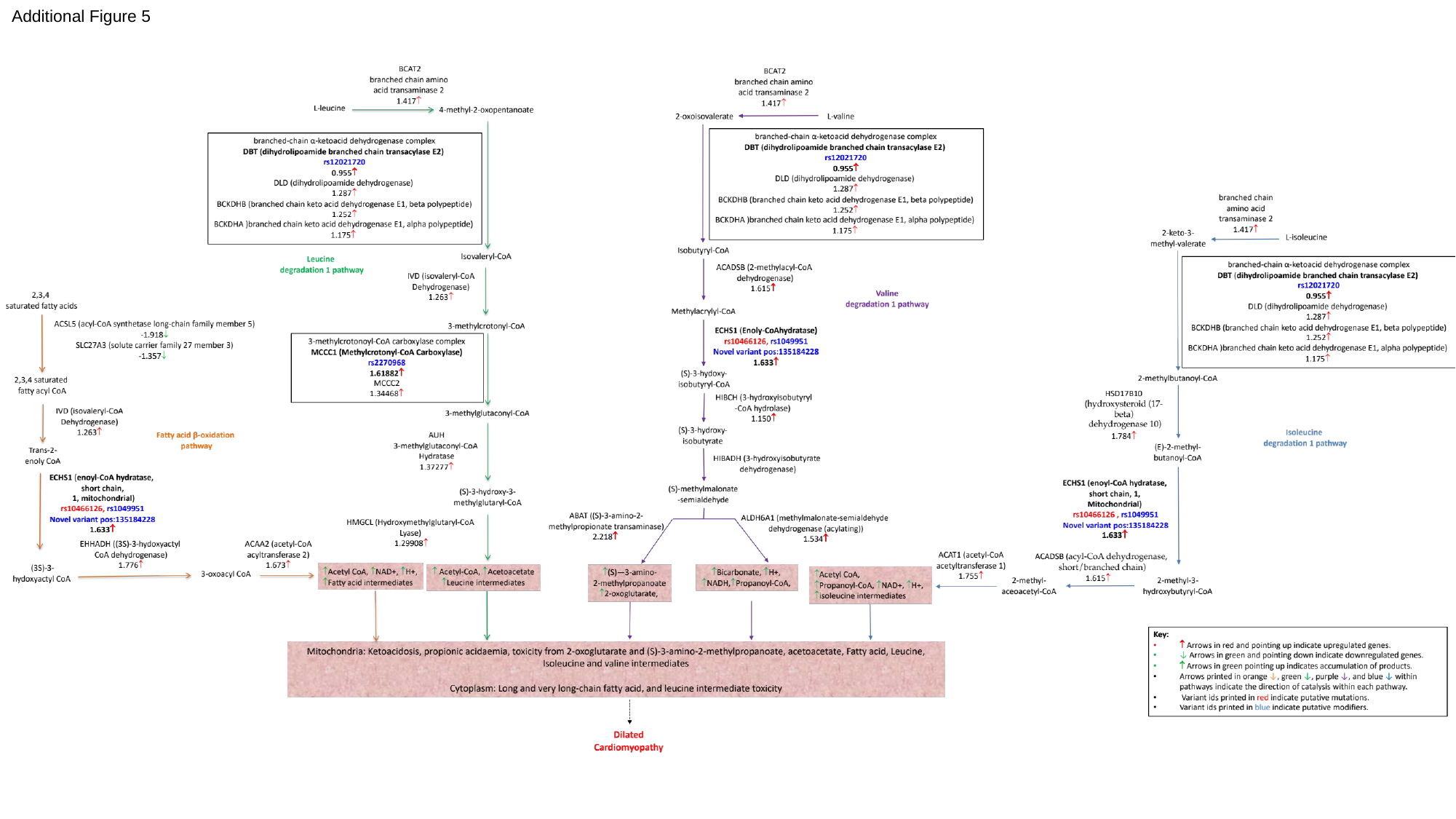

Additional Figure 5

Supplement: Supplementary file 6 — Figure S5. Proposed schematic: Dysfunction in the mitochondria. Legend: Arrows (red) pointing up represent up-regulated genes and arrows (green) pointing down, down-regulated genes. Printed in blue indicates putative modifiers and in red mutations. In bold print indicates genes carrying a variant. Orange arrows indicate the flow of catalysis in the fatty acid beta-oxidation pathway. Green arrows show the flow of catalysis in the Leucine degradation 1 pathway. Purple arrows show the flow of catalysis in the Valine degradation 1 pathway. Blue arrows indicate the flow of catalysis in the Isoleucine degradation 1 pathway. In boxes at the end of each pathway are proposed products of each pathway. (PPTX 583 kb) [file 12920_2018_439_MOESM6_ESM.pptx]
