# Supplementary material for: “Omics” data integration and functional analyses link Enoyl-CoA hydratase, short chain 1 to drug refractory dilated cardiomyopathy
Source: BMC Med Genomics. 2018 Dec 12;11:110. doi: 10.1186/s12920-018-0439-6 (PMC6292014; doi:10.1186/s12920-018-0439-6)
Supplement: Supplementary file 5 — Figure S4. Pathways associated with the ECHS1 gene. (PPTX 849 kb) [file 12920_2018_439_MOESM5_ESM.pptx]

## Slide 1
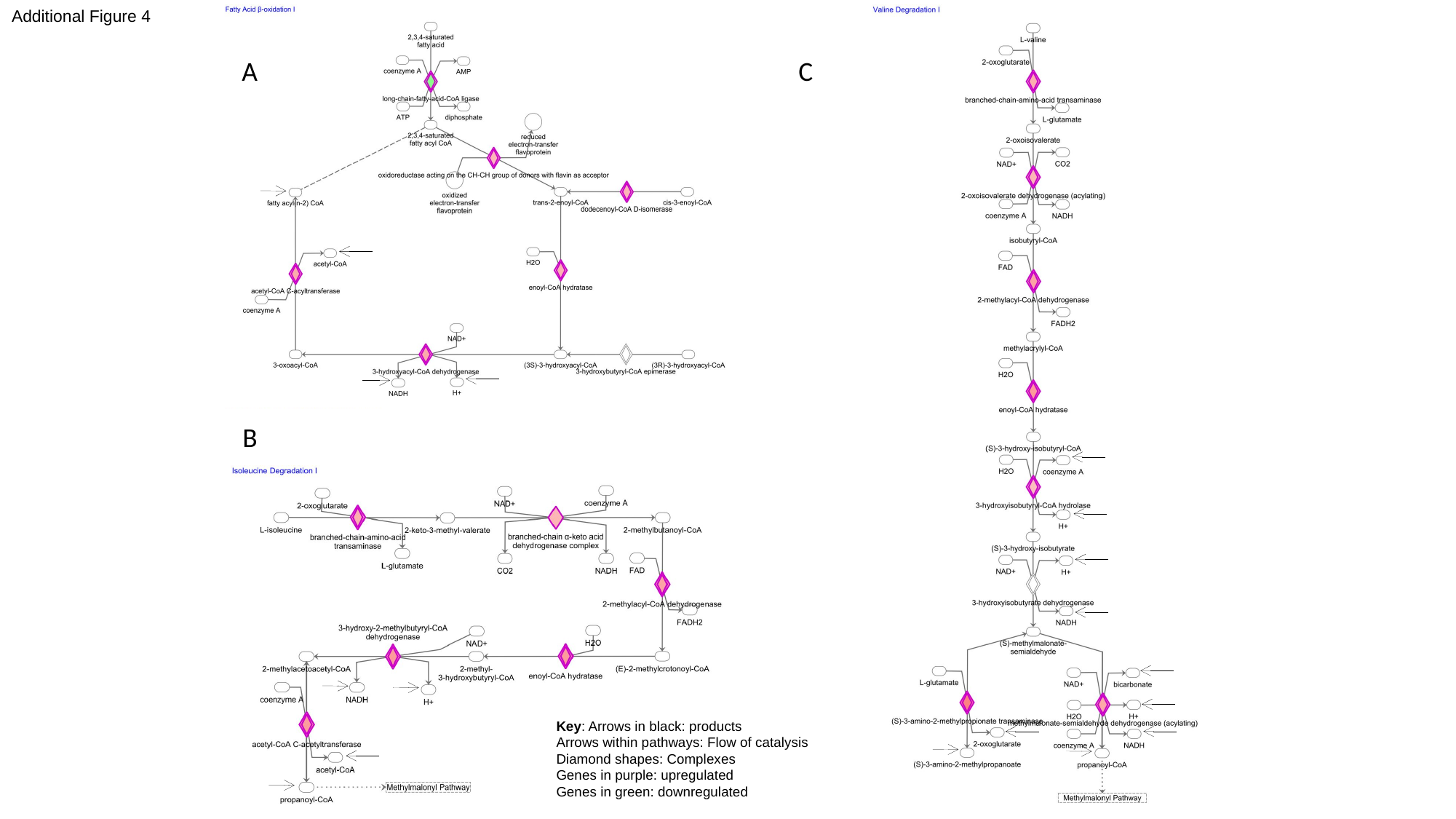

Additional Figure 4
A
C
B
Key: Arrows in black: products
Arrows within pathways: Flow of catalysis
Diamond shapes: Complexes
Genes in purple: upregulated
Genes in green: downregulated
